# Formulation Matters: The Overlooked Engine of Stability and Success in Antibody–Drug Conjugates

**DOI:** 10.3390/ph19030393

**Published:** 2026-02-28

**Authors:** Letícia Torres-Dias, Erik Moore, Surabhi Shukla, Alekha K. Dash

**Affiliations:** Department of Pharmaceutical Sciences, School of Pharmacy and Health Professions, Creighton University, 2500 California Plaza, Omaha, NE 68178, USA; leticiatorresdias@creighton.edu (L.T.-D.); erikmoore@creighton.edu (E.M.); surabhishukla@creighton.edu (S.S.)

**Keywords:** antibody–drug conjugates, formulation stability, linker–payload, excipient optimization, solid-state stabilization, targeted therapy

## Abstract

**Backgrounds:** Antibody–drug conjugates (ADCs) combine the specificity of monoclonal antibodies with the cytotoxic potency of drugs, representing a significant class of targeted cancer therapeutics. Despite their clinical success, formulation-related instability, rather than biological inefficacy, is a major contributing factor to setbacks in ADC development. This review examines the biochemical, physicochemical, and formulation factors that contribute to ADC stability, with a focus on excipient selection, conjugation site heterogeneity, and linker–payload reactivity. **Methods:** This comprehensive review was based on a selection of peer-reviewed mechanistic, analytical, and manufacturability studies on ADC stability. Our goal was to highlight formulation strategies, degradation pathways, and solid-state stabilization principles that affect the pharmacokinetics and therapeutic efficacy of ADC. **Results:** Results demonstrate how formulation variability including buffer composition, excipient choice, ionic strength, and lyophilization can directly affect payload release, linker cleavage, kinetics, and antibody conformation. It has been demonstrated that techniques, such as lyophilization with glass-forming matrices and the addition of surfactants, enhance stability against hydrolysis, oxidation, and aggregation. Developments in analytical characterization, such as real-time kinetic modeling and multi-attribute techniques based on mass spectrometry, have made quantification of degradation and bioactivity losses more predictable in ADC formulations. The connection between chemical stability and formulation outcomes is being redefined by new techniques, such as model-informed optimization and AI-driven design. **Conclusions:** ADC formulation is now a key component of molecular stability, clinical reliability, and regulatory compliance rather than a secondary consideration. By guaranteeing long-term stability, better pharmacokinetics, and improved therapeutic indices across next-generation designs, these approaches have the potential to revolutionize ADC development.

## 1. Introduction

The goal of combining the potency of cytotoxic molecules with the target specificity of monoclonal antibodies (mAbs) to selectively kill tumor cells while sparing healthy tissues gave rise to the therapeutic rationale for antibody–drug conjugates (ADCs) [1,2,3]. The first idea, based on Paul Ehrlich’s magic bullet theory, called for a carrier molecule that could transport deadly substances straight to target cells. However, inadequate conjugation chemistry, poor linker stability, and unpredictable pharmacokinetics continued to limit the realization of this novel concept for decades [4,5].

Due to unstable hydrazone linkers and poorly defined conjugation profiles, the first generation of ADCs in the late 1980s and early 1990s, such as Gemtuzumab ozogamicin (Mylotarg^®^), suffered from aggregation, off-target toxicity, and restricted therapeutic windows [6]. ADCs did not start to show significant clinical activity until more advanced conjugation methods, like maleimide–thiol chemistry, and cleavable dipeptide linkers, like valine–citrulline, were introduced [7,8,9]. With the approval of brentuximab vedotin (Adcetris^®^) in 2011 and trastuzumab emtansine (Kadcyla^®^) in 2013, ADCs became a recognized therapeutic modality [1,6,10]. These conjugated molecules demonstrated that if linker chemistry and formulation conditions were optimized, the controlled conjugation of a potent cytotoxic payload onto a clinically successful antibody could result in a drug delivery system with enhanced selectivity, quantifiable efficacy, and tolerable toxicity [4,5,9,11,12,13].

Since then, the field of ADC has grown at a never-before-seen rate, and their increasing clinical and industrial significance is highlighted by the exponential growth of ADC research and clinical translation [4]. More than two hundred candidates are presently undergoing active clinical development worldwide, with over twenty in Phase III (Table 1), and, currently, fourteen ADCs have received FDA approval to treat a variety of conditions (Table 2), including hematologic malignancies, gastric cancer, and breast cancer [2,3,4,14,15,16].

The concept of targeted cytotoxicity was confirmed by early implemented drugs like brentuximab vedotin (Adcetris^®^) and ado-trastuzumab emtansine (Kadcyla^®^), but they also highlighted the challenges of striking a balance between potency, stability, and safety [1,4]. This triad has since been improved, with each generation of ADCs gradually incorporating design control throughout the payload–linker and antibody domains. The most recent wave of approvals, such as telisotuzumab vedotin (Emrelis^®^), Datopotamab deruxtecan (Datroway^®^), sacituzumab govitecan (Trodelvy^®^), and trastuzumab deruxtecan (Enhertu^®^), represents the culmination of decades of biochemical optimization producing agents with better therapeutic indices and broader clinical applicability [15,19,20].

Clinical outcomes of antibody–drug conjugates are affected by at least two types of failure mechanisms: those resulting from target biology and those resulting from changes in the drug product itself. These mechanisms have different causes and require different development approaches despite the fact that they are commonly discussed together. Biological failure is a reflection of limitations related to target biology and disease context, such as dose-limiting systemic toxicity, heterogeneous antigen expression, resistance mechanisms, and insufficient clinical benefit despite target engagement [21]. Rovalpituzumab tesirine is a glaring late-stage example of biology-driven failure; when rovalpituzumab tesirine treatment was compared to standard therapy in the randomized Phase 3 TAHOE trial, overall survival was lower [22]. As a result, the program was discontinued based on tolerability and efficacy rather than the quality of the drug product [22]. Similarly, vadastuximab talirine was discontinued after the Phase 3 CASCADE trial because of excessive treatment-related mortality; this limitation was caused by systemic toxicity and exposure rather than instability attributed to the formulated product [23]. In contrast, physicochemical processes like aggregation, linker–payload deconjugation, and redistribution of DAR species are associated with formulation-related instability [24]. These processes can change pharmacokinetics and limit dosing strategies without compromising antigen specificity. Gemtuzumab ozogamicin (Mylotarg^®^) offers a documented clinical example where stringent handling guidelines and lyophilization were required due to limited solution stability, which had a direct impact on clinical implementation [25].

The evidence that the stability and physicochemical robustness of ADCs’ formulation are critical to their clinical performance has increased along with their chemical sophistication [3,26,27]. Formulation influences pharmacokinetics, biodistribution, and immunogenicity in addition to maintaining structural integrity during handling and storage [5,16,28,29]. Whether an ADC survives physiological and other stress conditions or fails to reach its target due to aggregation, deconjugation, or payload oxidation depends on the molecular interface between the antibody, linker, payload, and excipients [26,29,30,31].

ADCs have been redefined by this evolution as a dynamic system whose therapeutic success depends on how formulation, chemistry, process engineering, and molecular design come together to create a stable yet active structure [3,24]. This study focuses on formulation and stability problems specific to antibody–drug conjugates, which have a direct impact on product quality, pharmacokinetics, and clinical translation. The focus is on formulation-related stresses, stabilization techniques, and physicochemical degradation pathways that influence the evolution of important quality attributes during development and lifetime management. The intended audience includes formulation scientists, analytical scientists, and translational developers. The emphasis centers around formulation-relevant mechanisms and analytical approaches that support rational decision making from early discovery to scale-up, rather than an exhaustive discussion of ADC target biology or clinical efficacy. This review seeks to close the gap between molecular design, pharmacological performance, and translational concerns. In doing so, it emphasizes the long-overlooked role of formulation factors in translating the ADC concept into a useful clinical tool.

## 2. Antibody–Drug Conjugate Elements

**Figure 1:**
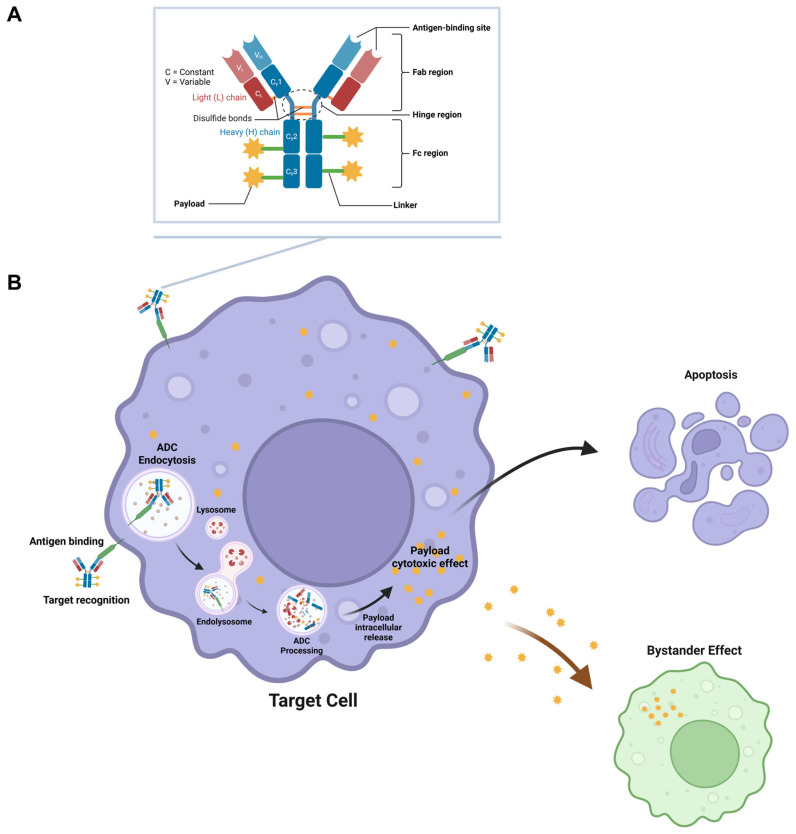
ADCs’ structure and mechanism of action: (**A**) depicts the structure of an ADC. The antibody contains two light chains and two heavy chains, and, inside of those, there are variable and constant regions. The chains are bound by disulfide bonds and a hinge that connects the fragment antigen binding (Fab) and Fragment Crystallizable (Fc) domains. The antigen-binding site is present at the Fab domain, and the cytotoxic payload is attached by a linker. (**B**) demonstrates the mechanism of action of ADCs. Following antigen recognition at the tumor cell surface, ADCs undergo receptor-mediated endocytosis and are intracellularly trafficked. ADC processing releases the intracellular payload, which induces cytotoxicity effects in the target cell, ultimately triggering apoptosis. When membrane-permeable payloads reach neighboring antigen-low or antigen-negative cells, they generate a bystander effect that enhances therapeutic efficacy in heterogeneous tumors. Created in BioRender. Torres Dias, L. (2026) https://BioRender.com/l80fies.

ADCs consist of three distinct elements: a monoclonal antibody that provides targeting specificity and a cytotoxic payload that offers cancer-killing effects, all attached by a linker that confers important stability, controlled release, and pharmacokinetics (See Figure 1A).

Antigen binding, internalization, intracellular trafficking, and payload release are among the biochemical and cellular processes that comprise an ADC’s mechanism of action, which is a multi-step molecular design (Figure 1B) [2,6,32,33,34,35]. When administered, the intact ADC circulates throughout the body, while formulation excipients, such as sugars, polysorbates, and amino acids, maintain it in a conformationally stable state [5,29]. The target antigen, expressed on the surface of tumor cells, such as HER2, TROP-2, CD79b, CLDN18.2, or CD123, is selectively bound by the antibody present [2,19,35,36,37,38,39,40]. Following binding, the ADC–antigen complex is endocytosed and trafficked through the endosomal-lysosomal system, where enzymatic pH or redox cues cause linker cleavage [9,40,41]. The released payload then exerts cytotoxic effects via mechanisms such as microtubule disruption, DNA damage, or topoisomerase inhibition, leading to apoptotic or mitotic cell death, which can also cause a bystander effect in neighboring cells when the payload diffuses across the target cell membrane to adjacent cells that may not express the target antigen at sufficient levels for ADC binding and internalization [11,34,40,42,43,44,45,46,47].

Table 1 presents ADCs currently undergoing Phase III clinical trials in the United States. From those, Trastuzumab deruxtecan (T-DXd), sacituzumab govitecan, datopotamab deruxtecan (Dato-DXd), mirvetuximab soravtansine, brentuximab vedotin, and trastuzumab emtansine (T-DM1) are previously approved ADCs currently being evaluated for expanded indications. In contrast, ifinatamab deruxtecan (I-DXd), sacituzumab tirumotecan (MK-2870/SKB264), tusamitamab ravtansine, and patritumab deruxtecan (HER3-DXd) represent new ADCs that have not yet received regulatory approval in the United States and are undergoing clinical evaluation.

### 2.1. Monoclonal Antibodies

The antibody component of antibody–drug conjugates (ADCs) is a monoclonal immunoglobulin that functions as the targeting moiety, offering specific identification of tumor-associated antigens and serving as the structural framework for the attachment of the linker to the payload. The antibody, which determines antigen specificity, half-life, biodistribution, and immunogenic potential, forms the foundation of the ADC structure [5]. Most clinical ADCs use humanized or fully human IgG1 antibodies due to the advantageous pharmacokinetic profiles and effector functions they provide [5,48,49]. High tumor selectivity, low normal-tissue expression, and effective internalization are characteristics of the ideal target antigen, such as HER2, TROP-2, or CD30 [2,49,50,51,52,53].

The engineering of monoclonal antibodies to exhibit a particular conjugation site is an essential strategy for ensuring formulation reproducibility and pharmacological consistency. Seki et al. [54] demonstrated that AJICAP^TM^-based site-specific conjugation produces homogenous ADCs with improved stability and predictable physicochemical behavior, indicating a strong correlation between downstream developability and antibody structure. Glycan-directed conjugation is another strategy to preserve Fc function while enabling site-specific payload attachment. Xue et al. [55] created JSKN003, a bispecific HER2-targeted ADC that kept biantennary glycan structures intact by using N-glycosylation-engineered conjugation. This design made the structure more hydrophilic, less likely to clump together, and more stable than thiol–maleimide constructs. It also achieved a high level of structural homogeneity and continued to work in trastuzumab-resistant phenotypes. These strategies demonstrate how antibody structure affects resistance modulation, formulation stability, and pharmacologic predictability in modern ADC design. These technologies ensure stable pharmacokinetics by reducing the instability and heterogeneity caused by random conjugation methods.

#### Monoclonal Antibodies Degradation Mechanisms and Analytical Tools

The primary pathways of instability that affect the monoclonal antibody component of ADCs are shown in Table 3. According to the reported data, thermal perturbation and hydrophobic surface exposure are common causes of conformational and colloidal instabilities, which can be significantly reduced by arginine salts, sucrose, and optimized histidine buffering [56,57,58]. Chemical changes continue to play a major role in ADC heterogeneity, and pH and temperature greatly influence deamidation and Asp isomerization, which are especially common in CDRs and hinge regions [59,60,61]. The oxidation of tryptophan and methionine residues can be slowed by antioxidants and light-resistant primary packaging. Because Met252 and Met428 are among these residues, ADC stability depends on them [62]. Antibody conjugation changes the hydrophobicity, tertiary structure, and molecular charge distribution of a molecule. If these changes are not properly controlled, they can speed up aggregation or degradation [26,63]. To evaluate post-conjugation stability and ensure uniform quality attributes, charge-variant profiling employing advanced analytical techniques, such as imaged capillary isoelectric focusing (icIEF) and high-resolution mass spectrometry (MS), has become essential [63,64].

Conformational instability is best supported by tools that directly report unfolding transitions, such as DSC or temperature-ramp fluorescence methods (DSF), because these assays quantify shifts in thermal stability that often precede aggregation under storage or processing stress [65]. When the dominant mechanism is colloidal instability driven by attractive protein–protein interactions or low solubility, DLS-derived interaction metrics and viscosity measurements provide a direct mechanistic link to the mitigation strategy (ionic strength adjustment, ArgHCl, or surfactants), as these methods quantify interaction propensity rather than only the endpoint of aggregate formation [66].

Aggregation requires size-resolving assays, and SEC remains the core stability-indicating method because it quantitatively separates monomers from soluble high-molecular-weight species and fragments [67]. Because interface-driven damage can generate particle populations that are not fully captured by SEC, subvisible particle methods (for example, flow imaging microscopy and light obscuration) are needed to demonstrate whether surfactants, reduced agitation, or optimized handling conditions suppress particle formation in a manner consistent with the proposed mechanism [68]. Charge heterogeneity and drift under formulation stress are most directly captured by icIEF and IEX, as these methods resolve changes in the charge-variant distributions that commonly arise from deamidation, isomerization, oxidation, or C-terminal Lys processing [69].

For chemical modifications, such as deamidation, Asp isomerization, and oxidation, peptide-mapping LC-MS provides the clearest linkage because it can localize and quantify site-specific changes, which is critical when mitigation strategies rely on narrowing pH windows, reducing light exposure, or modifying headspace oxygen [70]. Multi-attribute LC-MS approaches extend this capability by enabling targeted quantitation of multiple site-specific attributes within a single workflow while also supporting new peak detection during stability studies [70]. In practice, fragmentation and hinge cleavage are most convincingly supported when SEC is paired with an orthogonal size-based method, such as CE-SDS, because combining methods reduces the chance of overinterpreting chromatography artifacts as true fragmentation [65]. Finally, hydrophobicity-driven interactions and DAR-induced instability are best linked to assays that resolve drug load and hydrophobicity distributions, with HIC and RP-LC commonly used to track shifts in drug-loaded species that align with aggregation risk during concentration scaling or stress [71].

These improvements show that conjugation chemistry and antibody design are two parts of the same process that affect ADC formulation quality, not separate processes. Overall, these mechanisms highlight how crucial the antibody’s structural integrity is for maintaining ADC stability profiles.

**Table 3:** mAb-related instability pathways.

	Instability Pathways	Mechanisms	Mitigation Strategies	References
1	Conformational instability	Low thermal stability, unfolding transitions	Histidine or acetate buffers, sucrose/trehalose, arginine, controlled pH	[5,56,58]
2	Colloidal instability	Attractive protein–protein interactions, low solubility	Arginine-HCl, polysorbate 80/20, ionic strength adjustment	[5,56,57,72]
3	Aggregation	Partial unfolding, hydrophobic exposure, mechanical stress	Surfactants, trehalose, cyclodextrins reduced agitation, low temperature	[5,29,73,74]
4	Charge heterogeneity	Deamidation, isomerization, C-terminal Lys clipping	pH control, lower temperature, stabilizing buffers	[56,63,75]
5	Deamidation	Asn → Asp/isoAsp conversion (acid/base catalyzed)	pH 5–6, lower temperature, His buffering, lyophilization	[56,59,60]
6	Isomerization	Asp → isoAsp rearrangement	Mild pH, reduced heat/light exposure	[59,60,64]
7	Oxidation	Met/Trp oxidation by ROS or light	Antioxidants (methionine), amber vials, nitrogen headspace	[30,62,76,77]
8	Fragmentation	Acid/base hydrolysis, hinge cleavage	Stabilizing buffers, lower ionic strength, lyoprotectants	[56,78]
9	Hydrophobicity-driven interactions	Increased hydrophobic surface after payload addition	Arginine, glycine, cyclodextrins to reduce exposed hydrophobicity	[29,73,79,80]
10	DAR-induced instability	High DAR increases unfolding/aggregation	Lower DAR, site-specific conjugation	[81,82,83,84]

DAR, drug-to-antibody ratio; ROS, reactive oxygen species; Asn, asparagine; Asp, aspartic acid; isoAsp, isoaspartic acid; Met, methionine; Trp, tryptophan; His, histidine; Arginine-HCl, arginine hydrochloride.

### 2.2. Linkers

**Figure 2:**
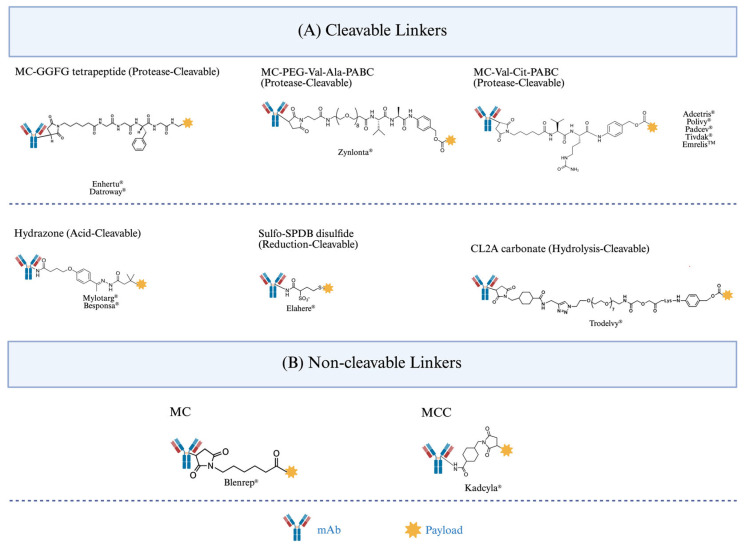
Classification of linker chemistries employed in currently FDA-approved antibody–drug conjugates: (**A**) cleavable linkers and (**B**) non-cleavable linkers. mAb, monoclonal antibody; MC, maleimidocaproyl; GGFG, glycine–glycine–phenylalanine–glycine; PEG, polyethylene glycol; Val, valine; Ala, alanine; PABC, para-aminobenzyloxycarbonyl; Cit, citrulline; sulfo-SPDB, sulfonated N-succinimidyl-4-(2-pyridyldithio)butyrate; SPDB, N-succinimidyl-4-(2-pyridyldithio)butyrate; CL2A, carbonate linker 2A; MCC, Maleimidomethyl Cyclohexane Carboxylate. Created in BioRender. Torres Dias, L. (2026) https://BioRender.com/eqlsvme.

The linker is the molecular hinge that connects the antibody and the payload (Figure 2). It controls how quickly the drug is released and how stable it is. To cause cytotoxicity, it must be able to promptly cleave within the target cell while staying intact during systemic circulation [9,41]. Cathepsin B can enzymatically cleave traditional linkers like valine–citrulline and valine–alanine, which are now seen as standards for Monomethyl Auristatin E (MMAE)-based ADCs [8,85,86]. On the other hand, non-cleavable linkers rely on the total lysosomal degradation of the antibody, which releases payloads as stable drug–amino acid conjugates with changed pharmacology [9,87].

Chemical changes to linkers can help improve pharmacokinetics and lower off-target toxicity. Watanabe and colleagues [7] created exo-cleavable linkers with hydrophilic motifs that made ADCs more stable in plasma and less likely to clump together. When comparing Exo-linkers and linkers in Exatecan-based ADCs, it was found that hydrophilicity led to lower non-specific uptake and better drug-to-antibody ratio (DAR) uniformity [8]. Similarly, Su and Zhang [41] showed how steric hindrance and the placement of disulfides in linkers change the kinetics and stability of payload release.

Multifunctional systems that can respond to stimuli or diagnosis have opened new avenues for linker design. To develop theranostic ADCs, Xiao and colleagues [88] integrated fluorogenic probes into peptide linkers, achieving concurrent cytotoxicity and in vivo imaging. Computational methods like Linker-GPT, which use AI to make new linker structures that work best for hydrophilicity, reactivity, and synthetic viability, have made the landscape even bigger [89]. These developments demonstrate that data-driven formulation design, in which linker chemistry is tailored to both molecular functionality and manufacturability, has shifted development away from empirical selection.

Certain chemical and biological processes determine linker–payload reactivity, and formulation conditions significantly influence their kinetics [12,41,90,91]. During storage or circulation, these kinetics determine whether payloads are released prematurely via off-target routes or primarily after target-mediated internalization [41]. Therefore, both formulation-dependent control of hydrolysis, exchange, reduction, and oxidative degradation activities at the linker–payload junction and the intrinsic design of the linker affect off-target payload release [41].

For cleavable linkers (Figure 2A), formulation conditions directly influence the rate of the chemical processes that cause payload release [24,27,41,92,93]. Cleavage of acid-labile linkers, such as the hydrazone linkers found in gemtuzumab ozogamicin (Mylotarg^®^), occurs via proton-catalyzed hydrolysis [93]. Water activity, buffer composition, and buffer capacity are crucial factors in determining whether hydrolysis occurs early in storage or in acidic intracellular compartments, because reaction rates increase as pH decreases [93,94]. Consequently, poor pH regulation or excessive moisture content can promote hydrolysis and lead to premature payload release.

The cleavage kinetics of protease-cleavable peptide linkers, such as the Val-Cit linkers employed in brentuximab vedotin (Adcetris^®^) and polatuzumab vedotin (Polivy^®^), are determined by peptide bond accessibility and molecular mobility [93,95,96]. Although these linkers are intended for fast enzymatic cleavage in lysosomes, formulation conditions that improve molecular flexibility, such as greater moisture content, liquid formulations, or higher temperature, reduce the kinetic barriers to peptide bond hydrolysis [41,94,97]. Off-target potential may increase in these situations due to accidental cleavage that may happen during storage or circulation.

Payload release from reducible disulfide linkers occurs via thiol–disulfide exchange processes with redox-sensitive kinetics [41,42,43,44,45,46,47,48,49,50,51,52,53,54,55,56,57,58,59,60,61,62,63,64,65,66,67,68,69,70,71,72,73,74,75,76,77,78,79,80,81,82,83,84,85,86,87,88,89,90,91,92,93,94,95,96,97,98,99]. With inadequate formulation control of redox conditions, trace thiols, excipient contaminants, and buffer composition can all change exchange rates, enabling reduction outside of the target cell [98]. Regardless of antigen binding, accelerated reduction permits systemic exposure and early payload release [98].

Non-cleavable linkers (Figure 2B), such as the thioether linker found in trastuzumab emtansine (Kadcyla^®^), do not rely on programmed cleavage to release payloads [82]. Instead, off-target release results from slower formulation-driven chemical breakdown processes [82,94]. The primary mechanism is oxidative stress, which could occur when peroxide species produced during surfactant degradation chemically alter the linker–payload junction or neighboring amino acid residues [30,31,100,101]. Despite being slower than enzymatic or hydrolytic cleavage, these cumulative kinetics over the course of the shelf life may alter drug-to-antibody ratio distributions and raise the amount of free payload in circulation, which could eventually lead to off-target exposure [100].

The linker classes depicted in Figure 2 all share a basic principle: off-target payload release is determined by formulation-dependent reaction kinetics operating based on diverse chemical mechanisms. Controlling pH, moisture content, buffer composition, redox environment, and excipient stability is thus critical to ensuring that payload release is linked to the desired biological trigger rather than unexpected chemical pathways.

#### Linker Degradation Mechanisms and Analytical Tools

The instability mechanisms specific to cleavable and non-cleavable linkers are shown in Table 4. Maleimide deconjugation remains one of the most well-understood processes, and direct evidence suggests that hydrolyzed maleimides or engineered conjugation sites significantly reduce thiol exchange with serum albumin [91,102]. Premature cleavage by glutathione or proteases is another major clinical issue [7,82]. Stability can be greatly increased while preserving cytotoxic release efficiency by modifying the hydrophobicity, steric bulk, or self-immolative spacer of the linker [7,14,94,97]. Studies on forced stability have demonstrated that reducing oxygen exposure or adding antioxidant excipients can prevent oxidative degradation of linker components [76]. The linker component can control the overall physicochemical behavior of ADCs, including their solubility and tendency to adhere to each other.

Linker liabilities often manifest as species redistribution rather than a simple loss of potency, so analytical mapping must focus on assays that directly measure changes in conjugation state and conjugated species’ integrity [24]. For maleimide hydrolysis and retro-Michael exchange, the most direct evidence is preservation of the conjugated species’ profile and suppression of exchange-related transfer reactions, which is why stability-oriented maleimide designs are commonly evaluated using orthogonal LC-MS and drug load distribution assays that can detect small but systematic shifts in conjugation [104]. Because these mechanisms often present as DAR drift and altered hydrophobicity distributions, HIC and RP-LC provide practical stability-indicating readouts that connect “exchange suppression” to a measurable retention and peak pattern consistent with stable drug loading [71].

Premature cleavage of protease-sensitive or acid-labile linkers requires analytics that can detect both the changing conjugated population and the appearance of released payload, as cleavage can occur without an immediate collapse of the monomer by SEC [24,82]. For this reason, the most mechanistically aligned evidence includes LC-MS/MS assays for unconjugated payload and catabolites in relevant matrices, paired with intact or subunit LC-MS to confirm whether linker-associated mass changes occur in parallel with payload release [117,118]. Oxidation of linker moieties is likewise best supported by chemical identity methods, as oxidation may not strongly shift size profiles until late and peptide-mapping LC-MS or MAM-style workflows provide a direct readout of oxidation signatures where they can be localized and trended during stability studies [70].

When the linker–payload region increases hydrophobicity and promotes self-association, SEC is needed to quantify soluble aggregates, but colloidal assays strengthen the mechanistic linkage by showing whether hydrophilic spacers or PEGylation reduce attractive interactions before aggregates appear [65]. In cases where steric constraints and linker length influence processing outcomes, the formulation-relevant analytical connection is typically indirect and should focus on whether the design change reduces heterogeneity in conjugated populations and yields more stable species distribution under stress, which can be confirmed using drug load resolving chromatography and LC-MS-based characterization rather than relying on a single endpoint assay [24,95,119].

### 2.3. Payloads in ADCs

The payload is the key effector component that determines the ADC’s potency and therapeutic index. Payloads in ADCs are potent cytotoxic drugs. Microtubule inhibitors (auristatins maytansinoids) [41,120], DNA-damaging agents (PBD dimers, calicheamicins) [121,122], and topoisomerase I inhibitors (DXd, Exatecan) [8,10,123] comprise the three most widely used payload classes. Each class has unique stability and toxicity factors that determine the formulation strategy. Payloads are chosen for their extreme cytotoxicity, but, because of their picomolar IC50 values, their chemical reactivity frequently necessitates careful control during conjugation and release [42,81].

Recent research indicates that structural modifications can increase the stability and safety of payloads. Wang et al. [86] showed that ionized Cys–linker–MMAE constructs increase plasma stability, decrease bystander effects, and maintain cytotoxic potency. Monomethyl auristatin F (MMAF) halogenation has been demonstrated to change conformational equilibria toward physiologically active forms without reducing potency [124]. Using mutasynthetic methods, novel ansamitocin analogs with modifiable substitution patterns have been created, leading to the sustainable synthesis of potent but chemically stable payloads [125].

One significant advancement is the use of a single antibody scaffold in multi-payload systems that deliver complementary drugs. Sun et al. [126] presented the Synergistic Payload–Antibody Ratiometric Conjugate (SPARC), a modular architecture that enables programmable drug combinations with synergistic antitumor effects while avoiding resistance. Polyamide–PBD hybrids, which limit DNA crosslinking, are another novel approach that reduces systemic toxicity [127]. Collectively, these instances demonstrate the increasing complexity of payload formulation as an integrated field that includes drug product engineering, molecular pharmacology, and synthetic chemistry.

#### Payload Degradation Mechanisms and Analytical Tools

Table 5 lists the molecular degradation pathways that impact cytotoxic payloads. Many of the small-molecule toxins used in ADCs, especially auristatins and maytansinoids, are naturally photolabile, and stability is greatly enhanced by standard photoprotective techniques [5,24,128,129]. One of the main causes of ADC aggregation is the hydrophobic nature of payloads, and, for that, PEG chains and cyclodextrin inclusion complexes with hydrophilic linkers improve solubility and reduce clumping without compromising strength [73,106,107,112,130]. The observation that payload degradation through hydrolysis or oxidation depends on chemical class and storage conditions underscores the importance of antioxidants or better control of the microenvironment in formulations [24]. All of these findings indicate that the payload component demands as much formulation scrutiny as the antibody backbone.

Payload-centered instability often introduces risks that are not captured by classical mAb stability assays unless the analytical workflow is explicitly designed around the payload’s chemical reactivity and optical properties [24,129,138]. For photodegradation, the mechanistic link is strongest when forced light exposure is paired with orthogonal readouts that capture both chemical changes and downstream physical consequences, as recent ADC-focused work has shown that light can induce payload-dependent degradation signatures and be mitigated by protective containers and controlled light handling [129]. When light exposure is suspected to be clinically relevant for certain payload classes, container closure selection (for example, amber vials) can be justified using published photostability data that specifically evaluates mitigation via light protection in ADC drug product contexts [128].

Payload hydrophobicity-induced aggregation is most defensible when the analytical package connects the physicochemical driver to measurable changes in both interaction propensity and aggregate formation, which is why SEC is typically paired with complementary biophysical or chromatography-based measures that reflect hydrophobicity and species distribution [67]. Direct analytical comparisons across major payload classes have demonstrated that conjugation decreases thermal stability and that payload and linker properties influence hydrophobicity and stability behavior, supporting the use of DSC plus SEC and RP-based methods to characterize how payload chemistry relates to developability [139]. When charge heterogeneity is driven by ionizable payload features or payload–linker contributions to the global charge distribution, icIEF method development studies in high-DAR ADCs provide a direct justification for using icIEF as a stability-indicating tool that can separate protein-driven versus payload–linker-driven charge behavior [69].

For payload degradation via hydrolysis or oxidation, the most direct stability linkage is chemical identity analytics, with LC-MS approaches (including peptide mapping for the protein component and small-molecule LC-MS/MS for payload-related species) providing complementary views of “what changed” and “how much free or modified payload appeared” [70]. Residual unconjugated payload is a distinct quality risk because it can exist even when conjugated species profiles appear acceptable and therefore requires direct quantitation through sensitive small-molecule methods rather than inference from DAR profiling alone [117]. Together, these analytics allow for mitigation strategies, such as oxygen control, antioxidant selection, photoprotection, and purification approaches, to be validated by demonstrating not only preserved monomer and DAR distribution but also reduced formation of released or chemically modified payload species.

### 2.4. Molecular Determinants of ADC Instability

ADCs are chemically fragile macromolecules that are vulnerable to numerous degradation pathways (Table 3, Table 4 and Table 5), endangering their effectiveness, safety, and manufacturability despite their conceptual accuracy [28,140]. This results in a multifactorial stability challenge where unfolding, aggregation, oxidation, linker hydrolysis, and payload degradation occur through interconnected mechanisms [26,27]. The foundation of contemporary ADC development is now the understanding and control of these processes through formulation design.

#### 2.4.1. Chemical and Physical Instability Mechanisms

Chemical reactions at the protein–payload interface often initiate ADC degradation. Deconjugation, payload degradation, oxidation of amino acid side chains, and hydrolysis of linker bonds are common pathways. As a result, aggregation, fragmentation, and charge heterogeneity frequently develop as downstream effects of structural perturbations within the conjugate [21,25].

##### Oxidation

One of the most crucial instability triggers is oxidation, especially in liquid formulations containing surfactants. Although polysorbates like PS-20 and PS-80 are frequently used to prevent aggregation, they are also vulnerable to oxidative degradation, which produces reactive aldehydes, peroxides, and free radicals [25,26]. These reactive species can catalyze linker cleavage, destabilize tertiary structure, and oxidize methionine or tryptophan residues on the antibody. Weber and colleagues [25] presented a mechanistic analysis of this process. They discovered that the primary causes of polysorbate oxidation are light exposure and trace metal contamination. Complementary work by Zheng et al. [26] showed that contact with stainless-steel surfaces accelerates surfactant degradation via iron–histidine–hydroperoxide interactions, emphasizing the importance of equipment and material choice in formulation control. In contrast to a passive background process, these studies reinterpreted excipient degradation as an active cause of ADC instability.

##### Aggregation

Particularly in liquid formulations, aggregation is another well-known mechanism of instability. Schuster et al. [67] showed that buffer composition affects monoclonal antibody aggregation, with bicarbonate buffers providing better stabilization than phosphate buffers. Aggregation in ADCs is exacerbated by increased hydrophobicity following drug conjugation, particularly when payloads are connected to solvent-exposed residues [5,49]. Because a higher drug-to-antibody ratio increases surface hydrophobicity and intermolecular interactions, the degree of conjugation heterogeneity, as measured by the DAR, correlates strongly with aggregation propensity [57]. The simultaneous quantification of DAR, free-drug impurities, and aggregation is now possible thanks to analytical advancements such as size-exclusion chromatography with dual UV detection [68] and hybrid HIC-MS workflows [69], enabling accurate formulation monitoring.

##### Photostability

Another molecular factor influencing ADC degradation is photostability; according to Thiess et al. [70], auristatin-based ADCs are especially vulnerable to UV-induced photodegradation, which results in DAR heterogeneity and aggregation. They demonstrated that when shielded by suitable excipients or packaging systems, some topoisomerase payloads show enhanced light resistance. This emphasizes photostability as an undervalued but formulation-dependent factor that requires light protection during production and storage. These findings demonstrate that ADC instability is not caused by a single chemical liability but rather by a combination of excipient reactivity, environmental stress, and structural design.

#### 2.4.2. Structural and Conjugation-Related Determinants

Conjugation site heterogeneity impacts formulation stability by influencing the local chemical environment and solvent exposure of the linker–payload moiety [81,141]. In randomly conjugated ADCs, such as lysine or reduced cysteine-linked designs, payloads are attached at different points with varying accessibility and structural flexibility [116,142]. Due to that, individual conjugation sites are subjected to various local environments, which causes variations in the tendency to aggregate linkers, cleave, and deconjugate during handling and storage, particularly in formulation stress situations like high protein concentration, freeze–thaw cycling, or agitation [142].

Site-specific conjugation methods limit payload attachment to preset sites with more uniform solvent conditions and arrangements, hence reducing variability [116,118,143]. Engineered cysteine, enzyme-mediated, and glycan-directed conjugation methods provide ADCs with reduced drug-to-antibody ratio distributions and less surface exposure of hydrophobic payloads [37,118,137,143]. From a formulation standpoint, enhanced homogeneity directly translates into improved colloidal stability, lower aggregation tendency, and more predictable storage behavior, particularly at high concentrations, and should be evaluated alongside excipient selection and processing conditions during ADC development [27,92].

As mentioned earlier, more consistent conjugates with predictable stability profiles, produced through site-specific technologies such as AJICAP^®^ [48] or glycan-engineered conjugation [50], can help optimize formulation. Watanabe et al. [144] additionally demonstrated that even heavily loaded ADCs can maintain stability if the conjugation architecture is logically designed by combining orthogonal conjugation techniques to achieve dual-payload ADCs with DAR = 10 while maintaining structural integrity.

The chemistry between the linker and the payload is another important factor responsible for instability. Cleavable linkers offer controlled release, but they are susceptible to premature hydrolysis or enzymatic cleavage in circulation, which can cause systemic toxicity and potency loss [9,41]. Watanabe et al. [7] tackled this issue by creating the exo-cleavable linker, a glutamic acid-modified valine–citrulline derivative that improved plasma stability and reduced aggregation. Similarly, Su and Zhang [41] showed that disulfide and maleimide linkers have very different redox stability and release efficiency, highlighting the need for matched linker selection based on the chemical environment of the conjugation sites.

ADC stability is also determined by the physicochemical characteristics of the payload, such as hydrophobicity, charge, and reactivity. As mentioned earlier, more hydrophilic payloads like the ionized cys–linker–MMAE constructs show better solubility and reduced aggregation rates, whereas highly hydrophobic payloads like auristatins can encourage self-association and aggregation [86]. To fine-tune both reactivity and formulation behavior, halogen substitution has been investigated as a solution to change electronic distribution and conformation without changing potency [124]. Collectively, these structural and chemical improvements show that stability is an emergent characteristic of several molecular interactions necessitating coordinated design among the payload, linker, and antibody components.

#### 2.4.3. Role of Excipients and Formulation Environment

Excipients control antibody–drug conjugate stability by influencing protein structure, intermolecular interactions, and the chemical environment around linker–payload moieties during storage and handling [24,145,146]. Payload conjugation enhances hydrophobicity and disrupts charge distribution, increasing sensitivity to excipient selection in contrast to unconjugated monoclonal antibodies and, therefore, demanding a more complex approach rather than directly transferrable formulation techniques [24,79].

Stabilizing sugars and polymers, such as sucrose (Suc), trehalose (Tre), and dextran 40 (Dex-40), essentially limits structural mobility via preferential exclusion in solution and vitrification in a solid state [147]. Thermal unfolding experiments demonstrate that increasing sugar content causes antibody unfolding transitions to occur at higher temperatures and slows aggregation during accelerated storage, which is consistent with lower molecular mobility [147,148]. In lyophilized systems, trehalose and sucrose-containing formulations preserve higher monomer concentrations than sugar-free controls, corresponding with higher glass transition temperatures and lower solid-state mobility [147,149]. In contrast, polymeric excipients like Dex-40 increase solution viscosity in a concentration-dependent manner, reducing diffusivity but introducing manufacturability and infusion limits at higher concentrations [150].

Surfactants, most frequently polysorbate 20 (PS20) and polysorbate 80 (PS80), are used to prevent interfacial adsorption and agitation-induced aggregation by occupying the air–liquid and container interfaces [29,30,101]. Agitation tests reveal that low polysorbate concentrations significantly minimize subvisible particle production relative to surfactant-free formulations [30]. However, polysorbates degrade hydrolytically and oxidatively during storage, resulting in peroxides that correlate with enhanced oxidation of sensitive residues, as demonstrated by peptide mapping [30,62]. Comparative degradation tests show that PS80 degrades faster than PS20 under identical conditions, which is consistent with the unsaturated fatty acid chain’s increased oxidation susceptibility [62]. These findings establish a trade-off between interfacial stabilization and chemically induced degradation risk.

Strickley and Lambert [5] listed more than one hundred commercial antibody formulations and recognized sucrose, trehalose, histidine, and polysorbates as fundamental stabilizers. These excipients guard against unfolding, aggregation, and surface adsorption brought on by stress. Álvarez-Palencia Jiménez et al. [29] experimentally confirmed this idea, demonstrating that the type of protein and the contact material both affect a surfactant’s ability to provide protection. According to their ELIBAG system, an experimental setup similar to an ELISA assay but incorporating a medical infusion bag as the contact surface, developed to study protein adsorption on different materials, it was reported that polysorbate-20 and poloxamer 188 demonstrated distinct efficiency profiles in preventing protein adsorption on glass and medical-grade plastics. Because surface adsorption can result in dose loss and irreversible aggregation during infusion, these findings are crucial for ADCs.

Buffering systems, such as histidine (His), histidine hydrochloride (His-HCl), sodium phosphate (NaPhos), sodium citrate (NaCit), sodium succinate (NaSucc), succinic acid (SuccAc), MES, and Tris-HCl, regulate pH-dependent degradation pathways and influence antibody conformation through ion-specific and ionic strength effects [24,56,146]. Antibodies produced in histidine buffers typically exhibit slower aggregation during accelerated storage than those in phosphate buffers at matched pH and concentration, as assessed through size-exclusion chromatography [151]. This behavior has been associated with variations in protein–protein interactions and hydration [151]. Phosphate buffers have a high buffering capacity but also enhance ionic strength, which can cause aggregation and phase separation in high-concentration formulations [56,146,148,152]. Citrate and succinate buffers successfully maintain a mildly acidic pH, although they have been demonstrated to accelerate acid-catalyzed breakdown when compared to histidine systems under otherwise similar conditions [56,146,148,152]. Schuster et al. [153] demonstrated that buffer composition plays a significant role. It was shown that by adjusting ionic interactions, bicarbonate-based buffers reduce aggregation more effectively than phosphate systems. pH is equally essential, as low-pH environments promote antibody unfolding and deconjugation while high-pH environments speed up base-catalyzed linker hydrolysis [24,29]. Finding a pH window that simultaneously maintains antibody integrity and linker stability is therefore necessary for ADC formulation development; this window is generally between 5.0 and 6.5 [24].

The effects of buffer species are strongly related to ionic strength, which determines electrostatic screening between antibody molecules [24,154]. Measurements of the diffusion interaction parameter (kD) demonstrate that low-ionic-strength histidine buffers sustain antibody-specific interaction behavior, but salt addition compresses kD into an excluded-volume-dominated regime, indicating the loss of charge-mediated repulsion [151,154]. Lower sodium chloride concentrations resulted in better monomer recovery following 30 days of storage in a bispecific antibody produced in histidine buffer, but higher salt levels retarded recovery to monomeric species [146,151,154]. Substitution of sodium chloride with arginine hydrochloride at equal ionic strength resulted in comparable trends, indicating that overall ionic strength was the most important component under those conditions [146]. Chelating compounds, like EDTA, are used to sequester trace metal ions that promote oxidative degradation events [5,24,62]. Metal-spiking investigations reveal that low micromolar concentrations of transition metals greatly boost oxidation in antibodies and ADCs, but the addition of chelators reduces oxidation to baseline values [5,24,62]. However, excessive chelation might affect protein–excipient interactions and container–closure compatibility, necessitating careful optimization [155].

Lyophilization has become a popular stabilization technique, according to the review by Wen and colleagues [27], to stop degradation in aqueous environments; fourteen out of sixteen globally approved ADCs in their review are available as lyophilized powders. However, they anticipated that liquid formulations would be prioritized in the future due to patient convenience and ease of administration. Advancements in excipient selection and container compatibility, incorporating approaches to limit surfactant oxidation [30,31] and adsorption mitigation [29], will be necessary for liquid-state stability.

Taken together, these findings indicate that excipient selection for ADC formulations is a compromise between conflicting stabilization processes rather than a single ideal solution. Sugars and polymers decrease conformational mobility, surfactants reduce interfacial stress while increasing oxidative risk, buffers regulate pH while altering electrostatic contacts, salts adjust ionic screening, and chelators inhibit metal-catalyzed degradation. Integrating comparative excipient evaluation with interaction and stability metrics allows for reasonable aggregation control, linker–payload integrity maintenance, and off-target payload mitigation throughout the ADC shelf life.

##### Limitations of Current Stabilization Strategies

Stabilization cannot be optimized in isolation, because formulation and clinical usability are tightly coupled. Current stabilization strategies for antibody–drug conjugates improve product quality but also introduce practical limitations related to manufacturing, scale-up, and lifecycle management, which should be acknowledged explicitly [5,24,92,156]. One limitation is increased formulation complexity, as the use of multiple excipients or narrow formulation windows can improve physical or chemical stability but may reduce robustness during manufacturing operations, such as filtration, filling, or freeze–thaw handling [157]. Higher excipient concentrations can increase viscosity or introduce compatibility constraints, which complicates large-scale processing even when stability benefits are observed at small scales [157,158].

Lyophilization is frequently used to address solution instability, but it introduces clear manufacturability trade-offs [149,156,159]. Freeze-drying increases processing times, costs, and operational complexity and requires tight control of freezing and drying conditions to avoid collapse or heterogeneity [156]. Improved solid-state stability must be balanced against sensitivity to residual moisture, reconstitution variability, and the need for specialized equipment and process control [156]. These factors limit the universal applicability of lyophilization despite its stabilizing benefits. Drug loading and hydrophobicity represent another trade-off between stability, pharmacology, and manufacturability. Studies demonstrate that increasing drug load or hydrophobicity can worsen aggregation propensity and reduce solubility, even though higher drug loading may improve in vitro potency [79,160,161]. These effects complicate formulation development and may require additional process controls or alternative linker–payload designs to maintain acceptable product quality [27,92,160].

Finally, tighter storage conditions, light protection, or temperature control can reduce degradation risk but increase distribution complexity and cost [162]. Regulatory guidance on stability testing emphasizes that approved storage conditions and shelf life must be supported by data and remain feasible across the commercial supply chain, reinforcing the need to balance stability gains against practical implementation [162]. Overall, current stabilization approaches improve ADC quality but involve unavoidable compromises between stability, manufacturability, and operational feasibility. Explicit recognition of these trade-offs supports more realistic formulation strategies and aligns development decisions with downstream manufacturing and lifecycle requirements rather than prioritizing stability alone.

## 3. Formulation-Centered Explanations for Why Many ADC Candidates Fail in Clinical Development

**Figure 3:**
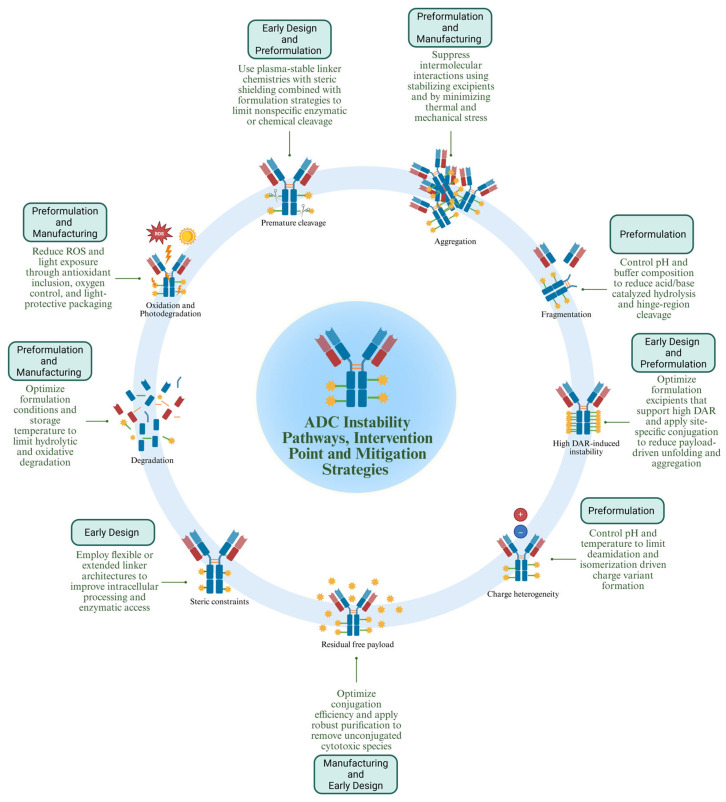
ADC instability pathways, intervention points, and mitigation strategies. The figure highlights major instability pathways, intervention points, and possible formulation and design-based approaches to control instability during ADC development and storage. ROS, reactive oxygen species; DAR, drug-to-antibody ratio. Created in BioRender. Torres Dias, L. (2026) https://BioRender.com/z8f21g2.

Antibody–drug conjugates have evolved from specialized tools for cancer treatment to a wide range of therapeutic classes. More than a dozen regulatory approvals and more than 400 clinical candidates have been reported; however, a high percentage of these programs are discontinued before or during late-stage development, often despite having valid targets and strong payloads [3,6,40,163,164]. Systematic clinical landscape analyses reveal that numerous approved ADCs fail due to unacceptable toxicity or insufficient efficacy at tolerated doses, emerging as significant reasons for termination, as the observed therapeutic window is considerably narrower than anticipated from preclinical data [6,40,164]. The difference between theoretical and clinical performance can be explained by the fact that ADCs are a complicated drug delivery system whose conjugation chemistry, payload properties, and excipient choices can work together in ways that either enhance or compromise stability (Figure 3) [92,165,166].

Figure 3 illustrates that instability in antibody–drug conjugates can be controlled through targeted intervention points applied at specific stages of development, and each intervention point can be associated with a degradation mechanism and structural domain.

At the antibody level, conformational instability and aggregation triggered by heat, agitation, or high concentrations can be mitigated mainly during preformulation through buffer optimization and excipient selection. Buffers and preferentially excluded sugars, such as sucrose or trehalose, stabilize the native fold, while arginine reduces protein–protein interactions that drive colloidal instability [65,152,167]. Surfactants are implemented at the drug product stage to prevent interface-induced aggregation [29]. Oxidative modifications are controlled through oxygen limitation, antioxidant inclusion, and nitrogen headspace management [76].

Linker instability is primarily addressed at the molecular early design stage, and maleimide exchange reactions are reduced through stabilized maleimide chemistries that undergo controlled hydrolysis, limiting retro-Michael exchange [168]. Premature protease cleavage is mitigated through linker design, including steric shielding and hydrophilic spacer incorporation [96]. Drug-to-antibody ratio heterogeneity, which contributes to aggregation and instability, is controlled through site-specific conjugation and DAR optimization during early design [82,169].

Payload-related intervention focuses on chemical protection and impurity control [129]. Photodegradation is mitigated through light-protective packaging, such as amber vials [129]. Oxidative and hydrolytic degradation are minimized through buffer microenvironment control and oxygen limitation [76]. Residual free payload, arising from incomplete conjugation or cleavage, is controlled during manufacturing through optimized stoichiometry and purification strategies, such as hydrophobic interaction chromatography, and quantified using LC-MS/MS assays [83,84,85,86,87,88,89,90,91,92,93,94,95,96,97,98,99,100,101,102,103,104,105,106,107,108,109,110,111,112,113,114,115,116,117,118,119,120,121,122,123,124,125,126,127,128,129,130,131,132,133,134,135,136,137,138,139,140,141,142,143,144,145,146,147,148,149,150,151,152,153,154,155,156,157,158,159,160,161,162,163,164,165,166,167,168,169,170,171].

Overall, instability is controlled through stage-specific intervention, molecular engineering during discovery, buffer and excipient optimization during preformulation, and environmental control during manufacturing and drug product development. Stability management in ADCs therefore depends on aligning each degradation mechanism with an appropriate formulation or design control applied at the stage where it is most effective.

When mapping approved versus discontinued programs, it is often found that aggregation, uncontrolled deconjugation, and unpredictable pharmacokinetics are major reasons for failure. All of these factors are greatly affected by the formulation environment and processing history [76,165,172,173].

According to several recent clinical studies, ADC development has a high failure rate; these studies usually blame target biology, linker–payload design, and therapeutic index for the failures, but they also consistently point out that ADCs are less stable than their parent monoclonal antibodies and often call for more careful dosage forms and handling [24,92,165]. Wen et al. [24] conducted a comprehensive analysis of globally available ADCs and demonstrated that by early 2025, 14 out of 16 approved ADCs were provided as lyophilized powders, specifically due to the shorter shelf life and greater instability of liquid formulations compared to solid-state approaches. When coupled with more extensive evaluations of biologics formulations, this trend strongly suggests that formulation-related instability is a latent factor contributing to ADC clinical failure and termination, even when dossiers predominantly indicate dose-limiting toxicity or insufficient efficacy at the clinical level [5,166,174].

Conjugation alters the balance between conformational and colloidal stability, rendering ADCs more fragile than the foundational IgG scaffold, a principal mechanistic focus in preclinical formulation research [74,76,102,173]. Even though spectroscopy shows that secondary and tertiary structures are mostly the same, thiol–maleimide and lysine-based conjugation routes both add hydrophobic drug–linker motifs to the surface. This makes the antibody CH2 (Constant Heavy 2 Domain) region less stable at elevated temperatures or when it is agitated, which speeds up the rate of aggregation [74,102,173]. Fluorescence, biophysical profiling, and differential scanning calorimetry all show that ADCs with higher DAR values, especially those in the DAR 6 to 8 subpopulations, are the first to convert into high-molecular-weight species. These species often represent irreversible structurally altered aggregates [74]. Numerous ADC-focused studies have encapsulated these findings, establishing a clear correlation among hydrophobic payloads, DAR distribution, and linker chemistry with aggregation, accelerated clearance, and a constrained therapeutic window, thereby positioning developability as a significant risk factor for clinical attrition [28,92,165].

In addition to hydrophobicity, several mechanistic studies demonstrate that conjugation sites, payload charge, and linker architecture can influence ADC stability in ways that formulations must either strategically leverage or mitigate [94,97,131,143]. Even though vcMMAF is less hydrophobic, tests with auristatin-based ADCs showed that adding a negatively charged vcMMAF payload lowered the isoelectric point and raised the charge heterogeneity compared to neutral vcMMAE. This made the particles come together more quickly under stress [131]. B Linker designs that incorporate PEG or saccharide moieties around hydrophobic payloads can improve solubility and pharmacokinetics while maintaining strong in vivo efficacy. This is because they partially hide their hydrophobicity and allow for stable DAR 8 constructs [106,112]. Site-specific conjugation platforms decrease heterogeneity and enable DAR 2 to 4 constructs with an enhanced therapeutic index. However, even in these instances, light-chain or Fc conjugation can locally destabilize domains and elevate aggregation propensity unless formulation conditions are meticulously optimized [55,115,116,143,171]. Overall, these studies show that many ADCs have a smaller stability margin than traditional mAbs. This means that even small mistakes in pH, excipient choice, or processing can have unwanted effects on patients [102,175,176].

Aggregation and chemical degradation are not merely cosmetic quality issues; several mechanistic studies demonstrate direct correlations between stress-induced alterations and off-target toxicity or loss of exposure, which are common reasons for discontinuing ADC programs [62,177]. Aoyama and colleagues [177] demonstrated that ADC aggregates, formed through stirring or thermal stress, facilitate Fcγ receptor-dependent uptake into target-negative cells, thereby significantly enhancing off-target cytotoxicity in FcγR-expressing lines. This effect was diminished by inhibiting FcγRs or inducing Fc silencing, highlighting aggregation as a mechanistic factor in off-tumor toxicity. Metal-catalyzed oxidation studies with IgG1 mAbs and their corresponding ADCs demonstrate that Cu(II) or Fe(II)-driven reactive oxygen species selectively oxidize Fc methionines and other residues. This results in increased carbonylation, fragmentation, and aggregation, as well as decreased binding to FcγRIIIa and FcRn, which is anticipated to impair effector function and reduce half-life [62]. Shelf life simulations using degrading polysorbate 80 as a reactive oxygen species source show that ADCs can also become unstable upon oxidation. In these tests, the conjugated constructs are often more affected than the parent antibody [76]. When clinical landscape analyses pinpoint dose-limiting toxicities and suboptimal pharmacokinetics as the main reasons for failure, mechanistic data indicates that formulation strategies that control aggregation and oxidation could convert borderline molecules into viable options, especially for agents with narrow therapeutic windows [24,28,164].

### 3.1. Regulatory Considerations

From a regulatory perspective, formulation stability is not limited to initial product development but extends across the full product lifecycle, including changes made during scale-up, post-approval manufacturing, and commercial supply [162,178,179,180,181,182]. Regulatory guidance frames stability as a central element linking comparability, shelf life assignment, and post-approval change control [162,181,182].

ICH Q5E defines comparability as a structured assessment to demonstrate that a product remains similar in quality, safety, and efficacy after a manufacturing or formulation change [182]. The guideline emphasizes a risk-based, stepwise approach, where the extent of data required depends on the nature of the change and its potential impact on critical quality attributes [182]. For formulation-related changes, such as buffer replacement, excipient level adjustment, or changes to lyophilization conditions, Q5E explicitly expects sponsors to evaluate whether these changes alter stability behavior, not only initial release characteristics [182]. In practice, this means that comparability packages often include side-by-side stability data for pre-change and post-change formulations under long-term and accelerated conditions [162,182]. Even when analytical comparability at release is demonstrated, stability differences over time can indicate a meaningful product change, requiring additional justification or mitigation strategies [182]. This regulatory expectation reinforces why formulation stability must be considered part of comparability rather than treated as a secondary attribute [162,182].

Shelf life assignment is governed primarily by ICH Q1A(R2), which defines the design of stability studies, required storage conditions, time points, and use of stability-indicating methods [162]. The guideline states that shelf life should be proposed based on data from primary stability batches and supported by a clear understanding of degradation pathways relevant to the formulation and container–closure system [162]. When long-term data does not yet cover the full proposed shelf life, Q1A(R2) allows commitments to continue stability studies post-approval, provided the available data justifies the proposed period [162]. ICH Q1E complements this by describing how stability data is evaluated to support shelf life proposals, including statistical approaches and conditions under which limited extrapolation may be considered [181]. Importantly, Q1E makes clear that extrapolation is not automatic and must be scientifically justified based on the consistency of degradation trends and the relevance of the analytical methods used [181]. Together, Q1A(R2) and Q1E establish that shelf life is a data-driven regulatory claim, not a formulation assumption, and that changes affecting stability behavior can require reassessment of the approved expiry period [162,181].

Post-approval formulation or process changes are regulated in the United States under 21 CFR 601.12 and corresponding FDA guidance, which classify changes based on their potential to affect product quality [179,180]. The FDA’s guidance on Chemistry Manufacturing and Controls (CMC) and changes to biological products explains that changes with a higher potential impact on stability, such as formulation composition or container–closure modifications, typically require more extensive reporting and supporting data [180]. ICH Q12 extends this concept by introducing tools for proactive lifecycle management, including the post-approval change management protocol (PACMP) [178]. Q12 explains that a PACMP allows sponsors to predefine how certain changes will be evaluated and reported, including the stability studies and acceptance criteria that will be used to demonstrate continued product quality [178]. FDA guidance on comparability protocols aligns with this approach, describing how a prospectively agreed-upon protocol can streamline regulatory review when post-approval changes are implemented [179]. These frameworks make clear that stability data is central to post-approval flexibility. Stability-indicating assays, acceptance criteria, and trending approaches established during development become the foundation for managing formulation changes throughout the product’s commercial life [162,178,179,180,181]. Discussing these regulatory expectations helps position formulation stability as a continuous regulatory obligation rather than a one-time development activity.

### 3.2. Solid-State Approaches and Excipient Selection to Improve ADC Stability

The formulation space itself presents additional pathways through which ADCs can fail, due to factors that are not primarily “biological.” Luo et al. [183] showed that therapeutic mAbs prepared at an acidic pH in dextrose-based diluents tend to form insoluble aggregates when mixed with serum. This occurs due to isoelectric precipitation of plasma proteins and the antibody at the blood–infusate interface. This highlights how even minor choices of diluents can lead to rapid aggregation in vivo. Typically, commercial antibody formulations are prepared within narrow pH ranges, often using buffers such as histidine or citrate and incorporating polysorbates as surfactants. However, these commonly used excipients are not completely inert, as their breakdown products can destabilize the formulations [5,56,166]. Polysorbate autoxidation and trace metal leaching from alloy containers, frequently used in pharmaceutical development, can trigger Fenton-like reactions, leading to oxidation, covalent cross-linking, and aggregation of monoclonal antibodies and antibody–drug conjugates (ADCs). These processes occur under stress conditions that might not reflect normal storage but can appear in exaggerated stability tests or poorly managed supply chains [62,76,77]. R. Dash and A.S. Rathore [159] compared repeated freeze–thaw cycles and lyophilization for trastuzumab and biosimilars, observing progressive increases in aggregation, charge variants, secondary structure changes, and potency loss across cycles. Lyophilization caused more pronounced alterations in binding than freeze–thaw alone. These results suggest that stressed solution formulations and poorly controlled lyophilization cycles can destabilize ADCs, potentially decreasing their therapeutic effectiveness or increasing immunogenicity concerns before intrinsic biological limitations occur.

Solid-state and lyophilized formulations are often used to address some drawbacks of solutions. Recent mechanistic studies reveal the specific conditions that ensure their benefits and identify situations where they may introduce new risks [24,92,149,174]. J. Ling et al. [174] present decades of water research, with sugar replacement and matrix vitrification models showing that disaccharides, such as sucrose and trehalose, can immobilize proteins within a glassy matrix, decrease molecular mobility, and stabilize structures across different motion regimes. These effects depend on strict control of residual moisture and the glass transition temperature. Cheng and colleagues [149] augment this mechanistic perspective with pragmatic guidance on lyophilized drug protein products, focusing on formulation design, container–closure selection, and cycle optimization. They highlight that high-concentration lyophilized systems necessitate meticulous excipient selection and process development to prevent collapse, microheterogeneity, or moisture-induced degradation. Wen et al. [24] analyzed marketed ADCs, revealing that the industry has standardized mainly lyophilization as the preferred method for commercial products. They also emphasized that the decision between lyophilized and liquid formulations must weigh long-term stability against the complexities of manufacturing and the challenges of reconstitution. Ji et al. [92] elucidate formulation strategies for ADCs, emphasizing that lyophilization can reduce aggregation and deconjugation in highly hydrophobic, high-DAR constructs, while noting that freezing and drying conditions, antioxidant use, and surfactant selection influence the extent of these benefits.

Lyophilization improves biopharmaceutical stability by turning labile aqueous formulations into solids, slowing deterioration due to reduced molecular mobility and diffusion [149,184]. Solid-state performance of protein-based products is heavily controlled by the physical state of the dried matrix and the formulation’s essential thermal properties, which are critical to both cycle design and long-term storage behavior [149].

ADC products commonly use solid dosage forms because conjugation can enhance solution instability relative to the parent antibody, making solid-state stabilization techniques more valuable [131]. A crucial stabilizing principle is to keep the dried product in a low-mobility amorphous regime, where the storage temperature is sufficiently lower than the system’s relevant glass transition behavior [156,184]. This idea limits accelerated thermal stressing in lyophilized products because temperatures above the product’s glass transition can undermine the significance of stability outcomes by shifting the material to a higher-mobility state [185]. This Tg-anchored framing is particularly critical for ADCs, as formulation screening and accelerated studies must avoid settings that artificially increase mobility-enabled pathways like aggregation or chemical changes at sensitive locations [185].

Moisture sensitivity is the second essential solid-state concept because leftover water plasticizes amorphous matrices and increases molecular mobility, accelerating physical and chemical degradation even when the designated storage temperature remains constant [184,186]. In lyophilized antibody formulations, residual moisture experiments reveal that stability trends cannot be deduced from Tg alone because increased residual moisture might reduce Tg while still producing formulation-dependent outcomes for aggregation and stability [187]. Zäh, M. et al. show an interesting approach connecting Tg with water activity as a stability-relevant descriptor, where water activity can be interpreted as a measure of how residual water interacts with the lyophilizate rather than just how much water is present [186]. Excipient selection influences Tg and moisture responsiveness by deciding whether the protein is encased in a protective amorphous matrix and how that matrix reacts to water uptake [184]. Jinghan L. and colleagues [167] reviewed the relationship of saccharides in freeze-dried protein formulations, identifying sucrose and trehalose as major stabilizers, and discussed their methods of preservation in dried states, arguing for their ongoing use as the default amorphous matrix formers in lyophilized biologics.

At the same time, moisture-induced variations in relaxation behavior were directly assessed in freeze-dried protein systems, including trehalose, demonstrating that matrix mobility and water–excipient interactions are not static after drying and remain stability-relevant during storage [188]. These similar solid-state principles are broadly compatible with what is observed in amorphous small-molecule systems, where Tg depression and moisture uptake cause materials to become more mobile, increasing the risk of physical instability [189]. As a result, for ADCs, a thorough lyophilization debate should treat Tg-anchored mobility control and moisture sensitivity as linked factors that must be co-optimized through formulation design, cycle development, and packing controls.

Solid-state studies also remind us that lyophilization is not a magic shield and can create pathways for degradation, which could harm clinical performance if not well understood. Valliere-Douglass et al. [152] assert that heat-stressed lyophilized mAbs and ADCs can undergo alterations in charge variants, potentially affecting stability and immunogenicity by forming covalent adducts between excipients or buffer components and protein side chains via condensation reactions favored in low-moisture environments. Confocal fluorescence microscopy has revealed protein–excipient microheterogeneity in dried formulations, indicating that standard industrial lyophilization conditions for IgG1 yield more uniform distributions, whereas particular spray-drying conditions may induce spatial segregation, potentially leading to local instability [190]. The importance of choosing excipient matrices with suitably elevated Tg and well-defined aging characteristics for the prolonged storage of protein solids is underscored by temperature-modulated and conventional DSC utilized on model amorphous drugs, which illustrate that physical aging beneath Tg leads to enthalpy relaxation and alterations in relaxation time constants [191]. Near-infrared spectroscopy techniques have been confirmed for the swift assessment of moisture content in lyophilized ADCs, offering a valuable means to regulate a factor that significantly affects solid-state stability and cake robustness [149,192].

More focused solid-state excipient strategies are emerging that address ADC-specific problems, such as interfacial aggregation and payload-induced instability. Research on cyclodextrins (CDs) with antibody–drug conjugates (ADCs) containing maytansinoid, auristatin, and fluorophore payloads indicates that hydroxypropyl-substituted CDs significantly diminish aggregation under shaking stress, presumably by concealing exposed hydrophobic surfaces or payloads. In contrast, sulfobutylether-β-CD exacerbates aggregation [73]. These results suggest that careful use of CDs, in either liquid or reconstituted form, could protect ADCs from stress during shipping and handling, a common point of failure in the manufacturing-to-clinic process. PEG-based linker designs, such as pendant mPEG24 chains attached to Val-Lys-PAB motifs, have enabled highly loaded DAR 8 ADCs with hydrophobic MMAE payloads to remain soluble, avoiding aggregation and exhibiting improved pharmacokinetics and tolerability in vivo [112]. Hydrophilic glycoside payloads, including MMAU and glucuronide-capped PBD dimers, have demonstrated the ability to reduce aggregation, enhance serum stability, and enable high-DAR constructs without sacrificing antitumor efficacy, thereby broadening the formulation design space for otherwise challenging payloads [130,193]. Formulation chemists and ADC researchers should see these new linker and excipient ideas as part of a bigger design plan. They should deliberately trade off linker hydrophilicity, DAR, and dosage form to keep molecules within a stability range that can be manufactured and used in the clinic.

Lastly, studies on clinical pharmacology and the development workflow show why these formulation-centered mechanisms are essential for success or failure at the trial level. Scaling analyses demonstrate that equivalent mg/kg dosing in both mice and humans results in comparable intratumoral ADC concentrations and tissue penetration, as tumor uptake is influenced by vascular permeability and surface area rather than body size [194]. Because ADCs need to reach specific intratumoral payload concentrations and the maximum tolerated dose in clinical settings often limits dosing, many agents operate only within a narrow exposure range. So, anything that reduces the delivered payload, such as an ADC that releases the payload too early while it is circulating, could make it less effective. Clinical reviews show that many ADCs that were discontinued failed because they could not destroy enough tumors at safe systemic doses. This problem gets worse when the formulation allows for payload loss, premature cleavage, or increased systemic off-target release [6,40,163,164]. Surveys of early-phase development timelines show that drug substance and drug product manufacturing decisions, such as selecting the proper dosage form and stability package, are among the longest steps between toxicology readouts and first-in-human dosing. This means that conservative, stability-driven formulation choices can directly affect how quickly and flexibly ADC programs can iterate or rescue borderline candidates [24,28,195]. When formulation mechanisms are considered in real clinical and drug development contexts, the challenges become clear. ADCs already pose risks due to their targets, payloads, and DAR, as well as formulation-centered instabilities, such as aggregation, oxidation, payload loss, and excipient reactivity. This means they can easily tip molecules from “barely viable” to “clinically untenable.”

In that sense, solid-state design and modern excipient strategies are not just ways to extend product life; they are also essential to widening the therapeutic window and preventing ADCs from being lost to toxicity or exposure loss due to instability. We believe that these strategies need to be used much earlier and more aggressively in the discovery and development of ADCs.

### 3.3. Analytical Advancements

Advanced analytical methods have been fundamental in identifying these formulation-driven failure modes and determining the solid-state design space that could prevent them. Two-dimensional liquid chromatography techniques that integrate size exclusion with reversed-phase chromatography, alongside native and denaturing mass spectrometry, have been employed to analyze size variants, DAR distributions, unconjugated drugs, and degradation products in ADC stability studies. These studies illustrate how minor modifications in conjugation or formulation conditions can impact both aggregate levels and payload integrity [78,84,196,197,198]. Hydrophilic interaction chromatography has enabled the complementary separation of glycoforms and polar variants while preserving the recovery of cysteine-conjugated ADCs [199]. Protein conformation assays, hydrogen–deuterium exchange MS, and DSC-based energetic analyses have been utilized to delineate local unfolding, CH2 destabilization, and domain-specific activation energies that correlate with aggregation during real-time and accelerated storage, enabling formulators to pinpoint susceptible domains and prioritize candidate conditions [58,75,76,143,200].

Multi-attribute mass spectrometry is often implemented as an LC-MS (liquid chromatography–mass spectrometry) peptide-mapping technique that allows for the focused monitoring of various site-specific product quality attributes as well as the identification of novel peaks using a single method [70,201]. However, regular implementation is limited by practical difficulties that go beyond analytical competence, such as method robustness, validation technique, and the operational requirements of maintaining constant performance over time [70,202]. Multi-attribute method (MAM) workflow requires controlled, reproducible sample preparation and LC-MS conditions to support consistent peptide-level readouts across batches [201,202]. Recent studies explicitly discuss sample preparation and LC-MS parameter troubleshooting for batch-style, higher-throughput applications and also demonstrate that MAM is frequently used to monitor specific modification classes, such as oxidation, deamidation, glycation, and glycosylation-related attributes, demonstrating both its scope and the importance of pre-defining which product quality attributes are targeted and how they will be interpreted [70,136,201,202].

A significant accessibility hurdle is the data-handling load imposed by peptide mapping LC-MS outputs, as well as the need for fit-for-purpose guidelines for attribute quantification and novel peak detection, which recent scientific and regulatory discussions indicate as critical concerns for quality control adoption [201,202,203]. In quality control contexts, MAM is described as not yet commonly recognized for Good Manufacturing Practice (GMP) batch release and stability testing due to limited experience and comfort with technical, compliance, and regulatory factors, prompting the publication of quality control specific advice [203]. This quality control focused advice expressly states that MAM deployment requires aligned approaches to compliance and regulatory objectives, rather than simply developing technical methods [203]. Regulatory-facing summaries from the FDA also frame MAM implementation around risk assessment, method validation, new peak detection, and comparison to conventional methods, indicating that routine deployment is dependent on demonstrating performance and control strategy fit rather than analytical depth alone [202,204]. Taken together, these arguments support establishing MAM as a high-value characterization and monitoring tool whose wider routine usage is limited by robustness, validation/interpretation frameworks, and quality control operational readiness, rather than sensitivity or selectivity restrictions.

#### Pharmacokinetics and Efficacy Implications

Analytical evaluation of antibody–drug conjugates is most useful when the results are interpreted in terms of the drug’s behavior in vivo. ADCs circulate as a mixture of intact conjugation, partially deconjugated antibody, and released payload, each with a unique contribution to pharmacokinetics and efficacy [41]. As a result, variations discovered through analytical methodologies can be directly connected to exposure, clearance, and tumor delivery, rather than simply serving as product quality descriptors [41,175]. One of the most obvious ties between analytics and pharmacokinetics is the drug-to-antibody ratio and hydrophobicity [79,82]. Preclinical and translational studies reveal that ADCs with higher drug content and higher hydrophobicity are cleared more quickly from circulation, resulting in decreased systemic exposure and a lowered therapeutic index despite higher in vitro potency [79,80,160]. Reverse phase (RP) and hydrophobic interaction chromatography (HIC) can be used to isolate ADC species based on drug load and present the relative abundance of each population, allowing in vitro DAR distribution to be used to predict clearance risk and exposure heterogeneity in vivo [71,171,205].

Linker stability establishes a second direct link between analytical data and efficacy. Linker chemistry governs how long the payload remains in circulation and how efficiently it is delivered at the target site [41,97]. Studies on ADC pharmacokinetics suggest that premature linker breakage reduces intact ADC exposure while increasing circulating free payload, which can diminish tumor delivery and increase systemic toxicity [41,97]. Analytical techniques, such as RP and LC-MS, that monitor drug load loss or the appearance of free payload help to determine if lower efficacy is caused by insufficient tumor exposure rather than a lack of intrinsic payload activity [41,97,206,207].

Pharmacokinetics is also influenced by aggregation and fragmentation, which are commonly measured using size-exclusion chromatography (SEC) [83,196,208,209]. Aggregated antibody species are known to clear faster due to improved immune recognition, lowering circulating amounts of active conjugate [146,176,208]. SEC results, in terms of potential clearance, enable aggregate formation found during storage or handling to be linked to lower exposure and, as a result, decreased efficacy.

Taken together, these examples demonstrate that analytical outputs, such as DAR distribution, linker integrity, and aggregation state, are not independent data but rather drivers of pharmacokinetic behavior and therapeutic effectiveness. The explicit relationship between analytical characterization, exposure, clearance, and payload distribution reveals how formulation and stability features can influence clinical efficacy across ADC programs.

## 4. Conclusions

Antibody–drug conjugates have evolved from an initial concept characterized by heterogeneous chemistry and limited therapeutic windows to one of the most advanced classes of targeted therapy in oncology. However, the field continues to encounter essential structural, physicochemical, and translational challenges that hinder the complete clinical potential of current platforms. A consistent theme emerges in the literature: despite the strong biological rationale for ADCs, as the primary obstacles to their success remain the instability of the antibody, payload, and linker, along with the complex interactions among these domains during production, storage, and physiological conditions [4,24]. Clinical data shows that even small increases in free payload levels or the creation of high-DAR aggregates can make toxicity much worse. On the other hand, losing conjugated species because of linker deconjugation or payload degradation makes efficacy worse [12,32,163]. A large number of ADCs that enter clinical trials do not make it past the first stage because of these weaknesses, even though their antigen targets and payload mechanisms are still helpful for treatment [210].

In the last ten years, better antibody engineering, site-specific conjugation linker chemistry, and payload design have helped with some of these problems, but the formulation landscape has changed more slowly [41,81,97,211]. Histidine buffers, sucrose or trehalose stabilizers, and polysorbate 20 or 80 surfactants are all examples of conventional excipient systems for monoclonal antibodies. These systems were first made for unconjugated IgG1 biologics, not for hydrophobic heterogeneous payload-bearing constructs, as many studies have shown [24,92,165]. Lyophilization has emerged as the primary method for ensuring long-term stability, particularly because it safeguards cargo from hydrolysis and oxidation. However, solid-state stabilization alone is insufficient to completely inhibit aggregation, thiol–maleimide exchange, payload photodegradation, or charge-dependent species drift, especially in high-DAR distributions [102,128,131,149]. The distinct physicochemical hazards linked to ADCs require a fundamentally more tailored methodology, despite the prevailing mAb-plus-payload paradigm in formulation practices.

The growing body of computational and analytical research supports this view. Data-driven frameworks like ADCNet have shown that the conjugation architecture, DAR distribution, and linker composition significantly affect degradation signatures and in vivo disposition. These behaviors can be predicted when enough molecular biophysical and stability data is combined [212]. Molecular generators and reinforcement-learning algorithms like Linker-GPT [89] show that the hydrophobicity, steric profile, and cleavage environment of linkers may all be improved quantitatively. This underscores that instability is a configurable aspect of the formulation, not an inherent characteristic of ADCs. Using historical data, you can change the excipient selection, freeze-drying settings, and surfactant systems to make stability margins bigger and failure modes smaller, as shown by AI-accelerated formulation science for different biopharmaceutical modalities, although these approaches currently function as decision support tools rather than replacements for experimental validation [213,214]. These changes mean that ADC formulation is now a significant part of determining the therapeutic index, rather than just one stage in the process.

Another rapidly growing area is the use of computer vision to assess the quality of lyophilized biologics. Automated imaging has demonstrated the ability to identify early particle development, cake collapse, container–closure integrity concerns, and other micro-architectural defects, facilitating real-time, non-destructive evaluation of lot quality [215]. Using AI-powered quality control tools in ADC manufacturing could help reduce the number of unstable mechanisms that arise after formulation decisions have been made. This is because lyophilization defects are closely linked to reconstitution variability, aggregation, and loss of potency [72,149,159].

In light of recent ADC research, it all points to the same conclusion: the primary constraint of ADCs is not solely their molecular architecture but rather the discordance between their inherent physicochemical susceptibilities and the excipient systems traditionally employed in their formulation. Current products reduce some degradation pathways, such as hydrolysis and oxidative stress, through lyophilization and amber packaging. However, they do not control others well enough, such as hydrophobic-payload-driven aggregation, maleimide rebridging, high-DAR species collapse, and multi-domain degradation in high-stress environments [24]. Because ADCs act more like complex chemical systems than antibodies with small-molecule appendages, the field is beginning to recognize that simply using mAb excipients is not enough. This gap is precisely what is driving growing interest in rational formulation design and the use of machine learning to discover patterns that human-guided empirical optimization struggles to identify.

In this respect, the direction outlined in this review, namely, the gradual transition toward ADC-specific formulation approaches informed by molecular degradation pathways and supported by emerging analytical and computational tools, represents an important opportunity for continued progress in the field. Framing ADC instability as a formulation and systems-level challenge, rather than exclusively a conjugation or chemistry problem, enables more targeted risk mitigation while remaining grounded in established development practice. Although advances in AI, solid-state science, and excipient design are still being integrated incrementally, their careful combination with validated formulation principles is likely to play a growing role alongside linker and antibody engineering in shaping the next generation of ADCs.

## 5. Future Directions

An interdisciplinary amalgamation of mechanistic formulation science, computational design frameworks, and digital quality control methodologies increasingly influence the future trajectory of ADC development. As the field progresses, innovative strategies are set to tackle the enduring instability pathways that current commercial formulations only partially alleviate [3,40,214]. Over the next years, several technological trends will converge to change how ADCs are designed, manufactured, and quality controlled. These developments will include generative AI systems, predictive pharmacokinetic modeling, real-time manufacturing analytics, and closed-loop optimization [24,216].

Predictive stability modeling that uses machine learning is one of the most critical opportunities. It has already been shown that algorithms may use structural descriptors, conjugation architecture, DAR distribution, and excipient composition to predict chemical degradation, aggregation, oxidation, and linker–payload cleavage for early ADC datasets [212,213]. As larger multi-modal datasets accumulate, these predictive systems will evolve from exploratory academic tools to industrial design engines capable of prioritizing high-stability constructs before chemical synthesis. Ultimately, such models may function as virtual preformulation platforms that guide excipient selection, stress testing design, freeze-drying parameters, and reconstitution protocols.

Generative molecular modeling, as demonstrated by the Linker-GPT framework, is poised to revolutionize linker and payload design by enabling exploration of chemical space beyond the capabilities of conventional medicinal chemistry [89]. By making more hydrophilic linker scaffolds, providing better steric protection, being released in a redox-controlled manner, or being less likely to exchange maleimide, these models can significantly reduce the formation of high-DAR aggregates or the premature release of payloads. When used with predictive formulation analytics, these tools could enable formulation and linker design to evolve together in fully integrated pipelines, rather than being optimized one after the other.

AI-enabled formulation optimization technologies are anticipated to become standard in biopharmaceutical development. Bayesian optimization digital twins of lyophilization processes and multi-parameter screening models will enable formulators to refine the extensive search space of buffer compositions, excipient ratios, surfactant combinations, and solid-state stabilization techniques [213,214,217]. Digital twin platforms can detect changes in cake shape, moisture content, or Tg during lyophilization and automatically adjust process settings in real time when used in manufacturing. These systems may mimic the impacts of changes in pH, humidity, temperature, freeze-drying cycles, and interactions between excipients and payloads. This helps in identifying strong design spaces that reduce aggregation and chemical degradation across DAR species [89,212,213,214,215,216]. A current application is to use machine learning to lessen the experimental burden of formulation screening by directing which formulations to test next [213]. According to Narayanan and colleagues [213], the method can find formulations that increase a biophysical stability parameter with far fewer experiments than traditional exploration of the same design space, using Bayesian optimization for biopharmaceutical formulation design. This suggests that in a real-world workflow, AI can assist in prioritizing a smaller group of buffer and excipient combinations for confirmatory stability tests, but standard stability and analytical readouts still play a role in the ultimate selection.

Predicting developability-linked liabilities, like aggregation rate and viscosity at high concentrations, that have a direct impact on formulation decisions is a second current application [158]. Lai and colleagues [158] provide machine learning models based on measured accelerated aggregation rates and viscosity for preclinical and clinical-stage antibodies at high concentrations, demonstrating how predictive models can help with earlier triage and lower late-stage reformulation risk. Related work on feature selection modeling reveals that aggregation behavior at high concentration may be predicted with few input features, demonstrating that machine learning can be effective when the targeted result is a specific, well-defined attribute related to formulation risk [218].

In contrast, future potential should be described with caution; the FDA emphasizes the need to define how AI/ML outputs will be evaluated, how models will be maintained over time, and how sponsors will ensure dependability when models are upgraded or retrained [219]. For ADC formulation development, it is reasonable to expect AI to eventually help integrate multi-modal datasets such as HIC or MS attributes, stability kinetics, and excipient libraries into “closed-loop” optimization, but these approaches are still most advantageous as enabling tools that narrow experimental searches rather than stand-alone decision makers.

Another critical area aims to include computer vision and anomaly detection methods with ADC quality control [214,215]. Automated imaging of lyophilized cakes has already been shown to be able to find early signs of problems that could lead to efficacy loss or aggregation later on [215]. When combined with complementary spectroscopic monitoring techniques, these imaging systems enable manufacturers to monitor product quality throughout freeze-drying, as well as during subsequent storage and distribution. This method is especially significant for ADCs, as minor physical flaws can generate heterogeneity reconstitution profiles, which can change DAR distribution and payload early release following administration. This is a concern that is especially important for cytotoxic modalities [140,210,215,220].

Future advancements will rely on the diversification of excipient systems, which will be thoroughly evaluated for ADC compatibility. Even though sucrose histidine buffers and polysorbates are standard in today’s products, there is not much new research on excipients [24]. Cyclodextrins, amino acid osmolytes, redox-stabilizing agents, and innovative hydrophilic polymer matrices constitute interesting yet inadequately investigated categories underpinned by robust mechanistic justifications from preliminary research in analogous biologics [24,73,76,106,107,221]. The next step in ADC formulation research will likely involve systematically mapping interactions between excipients and payloads, identifying steric encapsulation of hydrophobic domains, and testing how well the formulation protects against light- and oxidation-induced degradation. As computational methods improve, the field will be able to better match the characteristics of excipients to linker–payloads [89,212,213,214,215,216].

Another frontier is the integration of pharmacokinetic models with formulation design. The stability of the ADC in circulation significantly impacts DAR evolution, free-payload exposure, and off-target toxicity; hence, future ADC development will likely integrate predictive PK/PD models from the first stages [82,216]. A prominent example is Arrhenius-based kinetic modeling, which is used to forecast the long-term stability of monoclonal antibody compositions in solution [222]. Kuzman and colleagues [222] demonstrate that accelerated stability datasets (typically at intended, accelerated, and stress temperatures) can be combined with a first-order kinetic model to predict long-term behavior at the intended storage temperature, and the predictions are validated against long-term experimental data for up to 36 months. This provides a concrete decision framework for formulation work because formulations that exhibit a faster rate of change in attributes, such as aggregation or charge variants, under the same modeling approach can be deprioritized early, whereas formulations with slower kinetics can be advanced to longer studies [222].

A second example is “advanced kinetic modeling,” which is used across many industries and product types to produce stability estimates from short-term accelerated trials [223]. Huelsmeyer and colleagues [223] explain the use of Arrhenius-based kinetic models for stability forecasting, and they show that model predictions and real-time stability data agree for up to three years for products held at 2–8 °C or subjected to temperature fluctuations. Importantly for formulation decision making, they provide an explicit example of how using excessively harsh accelerated data resulted in poor predictions, whereas restricting the modeled temperature range improved prediction accuracy, demonstrating how kinetic modeling can guide the selection of “fit-for-purpose” stress conditions rather than relying on a fixed accelerated protocol [223].

For lyophilized formulations, a useful real-time kinetic example is to use isothermal microcalorimetry to quantify relaxation behavior quickly after freeze-drying and connect it with long-term crystallization during storage [224]. Groël et al. [224] used isothermal microcalorimetry to analyze amorphous freeze-dried formulations; they found that relaxation curves recorded within 12 h after lyophilization correspond with crystallization behavior evaluated for over 12 months, allowing for early detection of high-risk formulations. They also demonstrate formulation and process variables that directly inform decisions, such as the fact that polysorbate 20 accelerates sucrose crystallization and freezing has a strong effect on relaxation phenomena, implying that excipients and freezing conditions should be chosen rationally to reduce the risk of solid-state failure [224]. These examples demonstrate how real-time kinetic modeling aids formulation development; it converts early time-resolved data into interpretable rate parameters, assists in the selection of realistic accelerated conditions, and allows for early ranking of formulations and process options before long-term, real-time stability is achieved. These models, which are becoming more AI-driven, will show how formulation choices change conjugate half-life cleavage kinetics, lysosomal delivery, and bystander toxicity profiles [216]. These kinds of predictive platforms could help formulators develop formulations tailored to specific diseases or patient groups, ensuring that stability needs align with therapeutic windows.

Lastly, changes in the law will affect how these new technologies are used. AI-driven tools make it hard to understand and reproduce data scarcity models; therefore, regulatory frameworks will need transparent decision making backed by understandable AI architectures and thorough wet lab validation [214,215,216]. As experience grows, regulatory authorities may support hybrid workflows in which AI systems assist in decision making for formulation and production. At the same time, human specialists will assess whether mechanistic plausibility and clinical relevance are correct.

A common theme runs through all of these changes: the future success of ADCs will depend on a shift from antibody-based formulation tactics to a data-driven, mechanistic, and digitally enhanced approach. The combination of generative design, predictive stability modeling, adaptive production control, and improved quality assurance techniques has the potential to make ADC formulation science an entirely rational field. As these technologies converge, ADCs will be better able to deliver powerful, tailored cancer treatments that are safer, easier to manufacture, and more effective for patients. This transformation is necessary to make the next generation of therapeutically transformative ADCs possible, thereby fully realizing the potential imagined over decades of invention.

## Figures and Tables

**Table 1:** Current Phase 3 ADC clinical trials in the United States [17].

Name	Target	Indication	NCT Number(s)	Outcome
Trastuzumab deruxtecan (T-DXd)	HER2	Breast cancer	NCT03734029; NCT05950945; NCT03529110	Active/Recruiting
Trastuzumab deruxtecan (T-DXd)	HER2	Advanced or metastatic breast cancer	NCT04494425	Active
Trastuzumab deruxtecan (T-DXd)	HER2	Endometrial cancer	NCT06989112	Recruiting
Ifinatamab deruxtecan (I-DXd)	B7-H3 (CD276)	Small cell lung cancer	NCT06203210	Active
Sacituzumab tirumotecan (MK-2870/SKB264)	TROP2	Endometrial cancer	NCT06132958	Active
Sacituzumab tirumotecan (MK-2870/SKB264)	TROP2	Non-small cell lung cancer	NCT06074588	Recruiting
Sacituzumab tirumotecan (MK-2870/SKB264)	TROP2	Triple-negative breast cancer	NCT06841354	Recruiting
Sacituzumab govitecan	TROP2	Non-small cell lung cancer	NCT05609968; NCT05089734	Active
Datopotamab deruxtecan (Dato-DXd)	TROP2	Breast cancer	NCT05629585; NCT05374512; NCT06103864; NCT06112379; NCT05104866	Active/Recruiting
Datopotamab deruxtecan (Dato-DXd)	TROP2	Non-small cell lung cancer	NCT05687266	Active
Mirvetuximab soravtansine	FRα	Ovarian/peritoneal/fallopian tube cancer	NCT05445778	Recruiting
Brentuximab vedotin	CD30	Limited-stage Hodgkin lymphoma	NCT05675410	Recruiting
Brentuximab vedotin	CD30	Advanced-stage Hodgkin lymphoma	NCT02166463; NCT03907488	Active
Trastuzumab emtansine (T-DM1)	HER2	Breast cancer (adjuvant/combination)	NCT04457596	Active
Tusamitamab ravtansine	CEACAM5	Non-small cell lung cancer	NCT04154956	Active
Patritumab deruxtecan (HER3-DXd)	HER3	EGFR-mutant non-small cell lung cancer	NCT05338970	Active

ADC, antibody–drug conjugate; HER2, human epidermal growth factor receptor 2; HER3, human epidermal growth factor receptor 3; TROP2, trophoblast cell-surface antigen 2; FRα, folate receptor alpha; CEACAM5, carcinoembryonic antigen-related cell adhesion molecule 5; CD30, cluster of differentiation 30; B7-H3 (CD276), B7 homolog 3; EGFR, epidermal growth factor receptor.

**Table 2:** Current FDA-approved ADCs [18].

Product Name	Approval	Target	Indication	Excipients	CCS/ Shelf Life	DAR	Conj.	Linker	Payload
Gemtuzumab ozogamicin (Mylotarg^®^)	2000/2017 *	CD33	AML	NaPhos, NaCl, Dex-40, Suc, pH 7.5	Lyo, Amber glass, 60 Mo	~2–3	Lys	Hydrazone	Calicheamicin
Brentuximab vedotin (Adcetris^®^)	2011	CD30	Lymphoma	NaCit, Tre, PS80, pH 6.6	Lyo, Glass, 48 Mo	~4	Cys	MC-VC-PABC	MMAE
Trastuzumab emtansine (Kadcyla^®^)	2013	HER2	Breast CA	NaSucc, Suc, PS20, pH 5.0	Lyo, Glass, 48 Mo	~3.5	Lys	MCC	DM1
Inotuzumab ozogamicin (Besponsa^®^)	2017	CD22	ALL	Tris-HCl, NaCl, Suc, PS80, pH 8.0	Lyo, Amber glass, 60 Mo	~6	Lys	Hydrazone	Calicheamicin
Polatuzumab vedotin (Polivy^®^)	2019	CD79b	Lymphoma	SuccAc, NaOH, Suc, PS20, pH 5.3	Lyo, Glass, 30 Mo	~3.5	Cys	MC-VC-PABC	MMAE
Enfortumab vedotin (Padcev^®^)	2019	Nectin-4	Urothelial CA	His·HCl, Tre, PS20, pH 6.0	Lyo, Glass, 36 Mo	~4	Cys	MC-VC-PABC	MMAE
Trastuzumab deruxtecan (Enhertu^®^)	2019	HER2	Breast CA	His·HCl, Suc, PS80, pH 5.5	Lyo, Amber glass, 48 Mo	~8	Cys	Tetrapeptide	DXd
Sacituzumab govitecan (Trodelvy^®^)	2020	Trop-2	TNBC	MES, Tre, PS80, pH 6.5	Lyo, Glass, 36 Mo	~7.6	Cys	CL2A carbonate	SN-38
Belantamab mafodotin (Blenrep^®^)	2020/2025 **	BCMA	Myeloma	NaCit, Tre, EDTA, PS80, pH 6.2	Lyo, Glass, 48 Mo	~4	Cys	MC	MMAF
Loncastuximab tesirine (Zynlonta^®^)	2021	CD19	Lymphoma	His·HCl, Suc, PS20, pH 6.0	Lyo, Glass, 48 Mo	~2.3	Cys	Val-Ala-PABC	PBD dimer
Tisotumab vedotin (Tivdak^®^)	2021	TF	Cervical CA	His·HCl, Suc, PS20, pH 6.0	Lyo, Glass, 36 Mo	~4	Cys	MC-VC-PABC	MMAE
Mirvetuximab soravtansine (Elahere^®^)	2022	FRα	Ovarian CA	His, Suc, PS20, pH 6.0	Liq, Glass, 60 Mo	~3.4	Lys	Sulfo-SPDB	DM4
Datopotamab deruxtecan (Datroway^®^)	2025	Trop-2	Breast/ NSCLC	His, Suc, PS20, pH 6.0	Lyo, Glass, 36 Mo	~4	Cys	Tetrapeptide	DXd
Telisotuzumab vedotin (Emrelis™)	2025	c-Met	NSCLC	His, Suc, PS80, pH 6.0	Lyo, Glass, 36 Mo	~3–3.7	Cys	MC-VC-PABC	MMAE

ADC, antibody–drug conjugate; DAR, drug-to-antibody ratio; AML, acute myeloid leukemia; ALL, acute lymphoblastic leukemia; CA, cancer; TNBC, triple-negative breast cancer; NSCLC, non-small cell lung cancer; CD, cluster of differentiation; HER2, human epidermal growth factor receptor 2; BCMA, B-cell maturation antigen; TF, tissue factor; FRα, folate receptor alpha; c-Met, hepatocyte growth factor receptor; Nectin-4, nectin cell adhesion molecule 4; Trop-2, trophoblast cell-surface antigen 2; NaPhos, sodium phosphate; NaCl, sodium chloride; Dex-40, dextran 40; Suc, sucrose; NaCit, sodium citrate; NaSucc, sodium succinate; SuccAc, succinic acid; NaOH, sodium hydroxide; His, histidine; His·HCl, histidine hydrochloride; Tris-HCl, tris(hydroxymethyl)aminomethane hydrochloride; MES, 2-(N-morpholino)ethanesulfonic acid; Tre, trehalose; PS20, polysorbate 20; PS80, polysorbate 80; EDTA, ethylenediaminetetraacetic acid; Conj, Conjugation; Lyo, lyophilized; Liq, liquid; Mo, months; MMAE/F, monomethyl auristatin E/F; DM1/DM4, maytansinoid payloads; DXd, deruxtecan; SN-38, active irinotecan metabolite; PBD, pyrrolobenzodiazepine; MC, maleimidocaproyl; MCC, Maleimidomethyl Cyclohexane Carboxylate; VC, valine–citrulline; PABC, para-aminobenzyl carbamate; Val-Ala, valine–alanine; SPDB, N-succinimidyl 4-(2-pyridyldithio)butyrate. * Mylotarg^®^ was initially FDA-approved in 2000, voluntarily withdrawn in 2010, and re-approved in 2017 with a modified fractionated dosing regimen. ** Blenrep^®^ received accelerated FDA approval in 2020, was withdrawn from the U.S. market in 2022, and re-approved in 2025. All products are administered by intravenous (IV) infusion and stored at 2–8 °C.

**Table 4:** Linker-related instability pathways.

	Instability Pathways	Mechanisms	Mitigation Strategies	References
1	Maleimide hydrolysis/retro-Michael reaction	Thiol–maleimide adduct reversibility, exchange with serum albumin	Hydrolyzed maleimide to stable succinamic acid, site-specific conjugation minimizing exchange	[91,102,103,104,105]
2	Premature cleavage (acid/protease/glutathione sensitivity)	Overly labile dipeptide linkers (Val-Cit, PABC) in systemic circulation	Linker steric shielding; introducing hydrophilic spacers, modifying PABC to reduce systemic cleavage	[89,90,91,92,93,94,95,96,97,98,99,100,101,102,103,104,105,106,107,108]
3	Oxidation of linker moieties	Thioether or aromatic linker oxidation by ROS	Methionine, N-acetylcysteine, or histidine as sacrificial antioxidants, low-oxygen headspace	[76,109,110,111]
4	Hydrophobicity-driven aggregation from linker–payload region	Hydrophobic linkers increase antibody self-association	Hydrophilic PEGylated linkers decrease aggregation and improve solubility	[106,107,112]
5	Steric constraints and linker length issues	Short or rigid linkers impair internalization and enzymatic cleavage	Elongated or flexible linkers improve processing and reduce steric hindrance	[41,95,112,113]
6	mAb–linker interface instability	Cys reactivity, hinge region microenvironment affects conjugate stability	Site-specific conjugation stabilizes the interface	[81,105,114,115,116]

mAb, monoclonal antibody; ROS, reactive oxygen species; PEG, polyethylene glycol; Cys, cysteine; Val-Cit, valine–citrulline; PABC, *p*-aminobenzyl carbamate.

**Table 5:** Payload-related instability pathways.

	Instability Pathways	Mechanisms	Mitigation Strategies	References
1	Photodegradation	Payload chromophores can undergo UV-induced structural cleavage	Amber vials, minimize UV exposure, inclusion of photostabilizers	[24,128,129]
2	Payload hydrophobicity-induced aggregation	Highly hydrophobic payloads drive self-association and reduce solubility	Hydrophilic linker–payload design, PEGylation, cyclodextrin complexation improving solubility	[73,79,80,112,130]
3	Charge heterogeneity in payload region	Ionizable groups undergo protonation-state shifts, altering ADC pI	Buffer optimization controlling pH microenvironment, hydrophilic spacers reducing charge clustering	[56,63,131,132]
4	Payload degradation (hydrolysis/oxidation)	Maytansinoids: thiol-reactive degradation; auristatins: peptide bond hydrolysis	Antioxidants, low-oxygen headspace, temperature control	[76,130,133]
5	Residual unconjugated payload	Incomplete conjugation leads to free drug contamination	Optimized conjugation stoichiometry, purification through hydrophobic interaction chromatography	[116,134,135,136,137]

UV, ultraviolet; PEG, polyethylene glycol; ADC, antibody–drug conjugate; pI, isoelectric point.

## Data Availability

No new data were created or analyzed in this study. Data sharing is not applicable to this article.

## Abbreviations

The following abbreviations are used in this manuscript:

ADC	Antibody–Drug Conjugate
ADCNet	Antibody–Drug Conjugate Network
AI	Artificial Intelligence
Ala	Alanine
ALL	Acute Lymphoblastic Leukemia
AML	Acute Myeloid Leukemia
Asn	Asparagine
Asp	Aspartic acid
BCMA	B-Cell Maturation Antigen
CA	Cancer
CD123	Cluster of Differentiation 123
CD22	Cluster of Differentiation 22
CD276	Cluster of Differentiation 276
CD30	Cluster of Differentiation 30
CD33	Cluster of Differentiation 33
CD79b	Cluster of Differentiation 79B
CDRs	Complementarity-Determining Regions
CDs	Cyclodextrins
CH2	Constant Heavy 2 Domain
Cit	Citrulline
CL2A	Carbonate Linker 2A
CLDN18.2	Claudin-18
c-Met	Hepatocyte Growth Factor Receptor
Cu(II)	Copper(II) Ion
Cys	Cysteine
DAR	Drug-to-Antibody Ratio
DM1	Maytansine Derivative 1
DM4	Maytansine Derivative 4
DNA	Deoxyribonucleic Acid
DSC	Differential Scanning Calorimetry
DXd	Deruxtecan
EDTA	Ethylenediaminetetraacetic Acid
EGFR	Epidermal Growth Factor Receptor
ELIBAG	Enzyme-Linked Immunobinding Assay on Bags
Fab	Fragment Antigen-Binding
Fc	Fragment Crystallizable
FcRn	Neonatal Fragment Crystallizable Receptor
Fcγ	Fragment Crystallizable Gamma
Fcγrs	Fragment Crystallizable Gamma Receptors
Fe(II)	Iron(II) Ion
Flt3L	FMS-Like Tyrosine Kinase 3 Ligand
GGFG	Glycine–Glycine–Phenylalanine–Glycine
GPT	Generative Pre-Trained Transformer
GSDME	Gasdermin E
HER2	Human Epidermal Growth Factor Receptor 2
HER3	Human Epidermal Growth Factor Receptor 3
HIC-MS	Hydrophobic Interaction Chromatography–Mass Spectrometry
IC50	Half-Maximal Inhibitory Concentration
icIEF	Imaged Capillary Isoelectric Focusing
IgG1	Immunoglobulin G1
IV	Intravenous
Liq	Liquid
Lyo	Lyophilized
Lys	Lysine
mAb	Monoclonal Antibody
MC	Maleimidocaproyl
MCC	Maleimidomethyl Cyclohexane Carboxylate
MES	2-(N-Morpholino)ethanesulfonic Acid
Met	Methionine
Met252	Methionine 252
Met428	Methionine 428
MMAE	Monomethyl Auristatin E
MMAF	Monomethyl Auristatin F
MMAU	Β-D-Glucuronyl-Monomethylauristatin E
Mo	Months
mPEG24	Methyl-Polyethylene Glycol24
MS	Mass Spectrometry
NaCl	Sodium Chloride
NaOH	Sodium Hydroxide
Nectin-4	Nectin Cell Adhesion Molecule 4
NSCLC	Non-Small Cell Lung Cancer
PAB	Para-Aminobenzyl
PABC	Para-Aminobenzyl Carbamate
PBD	Pyrrolobenzodiazepine
PEG	Polyethylene Glycol
pH	Potential of Hydrogen
pI	Isoelectric Point
PS-20	Polysorbate 20
PS-80	Polysorbate 80
ROS	Reactive Oxygen Species
SN-38	7-Ethyl-10-Hydroxycamptothecin
SPARC	Synergistic Payload–Antibody Ratiometric Conjugate
STING	Stimulator of Interferon Genes
TF	Tissue Factor
Tg	Glass Transition Temperature
TLR4	Toll-Like Receptor 4
TNBC	Triple-Negative Breast Cancer
TROP-2	Trophoblast Cell Surface Antigen 2
Trp	Tryptophan
UV	Ultraviolet
Val	Valine
vcMMAE	Valine–Citrulline Monomethyl Auristatin E
vcMMAf	Valine–Citrulline Monomethyl Auristatin F

## References

[1] Senter P.D., Sievers E.L. (2012). The Discovery and Development of Brentuximab Vedotin for Use in Relapsed Hodgkin Lymphoma and Systemic Anaplastic Large Cell Lymphoma. Nat. Biotechnol..

[2] Ruan D.Y., Wu H.X., Meng Q., Xu R.H. (2024). Development of Antibody-Drug Conjugates in Cancer: Overview and Prospects. Cancer Commun..

[3] Wang R., Hu B., Pan Z., Mo C., Zhao X., Liu G., Hou P., Cui Q., Xu Z., Wang W. (2025). Antibody–Drug Conjugates (ADCs): Current and Future Biopharmaceuticals. J. Hematol. Oncol..

[4] Colombo R., Tarantino P., Rich J.R., Lorusso P.M., de Vries E.G.E. (2024). The Journey of Antibody–Drug Conjugates: Lessons Learned from 40 Years of Development. Cancer Discov..

[5] Strickley R.G., Lambert W.J. (2021). A Review of Formulations of Commercially Available Antibodies. J. Pharm. Sci..

[6] Gogia P., Ashraf H., Bhasin S., Xu Y. (2023). Antibody–Drug Conjugates: A Review of Approved Drugs and Their Clinical Level of Evidence. Cancers.

[7] Watanabe T., Arashida N., Fujii T., Shikida N., Ito K., Shimbo K., Seki T., Iwai Y., Hirama R., Hatada N. (2024). Exo-Cleavable Linkers: Enhanced Stability and Therapeutic Efficacy in Antibody-Drug Conjugates. J. Med. Chem..

[8] Watanabe T., Iwai Y., Shikida N., Stofleth J.T., Fujii T., Seki T., Shimbo K., Matsuda Y. (2025). Biological Evaluation of Cleavable Linkers in Exatecan-Based Antibody–Drug Conjugates: A Comparative Study of DXd and Exo-Linker Platforms. ACS Omega.

[9] Yang L., Ma J., Liu B., Li Y., Ma Y., Chen H., Han Z. (2025). Recent Advances in Peptide Linkers of Antibody–Drug Conjugates. J. Med. Chem..

[10] Okamoto H., Oitate M., Hagihara K., Shiozawa H., Furuta Y., Ogitani Y., Kuga H. (2020). Pharmacokinetics of Trastuzumab Deruxtecan (T-DXd), a Novel Anti-HER2 Antibody-Drug Conjugate, in HER2-Positive Tumour-Bearing Mice. Xenobiotica.

[11] Suzuki H., Nagase S., Saito C., Takatsuka A., Nagata M., Honda K., Kaneda Y., Nishiya Y., Honda T., Ishizaka T. (2024). Raludotatug Deruxtecan, a CDH6-Targeting Antibody-Drug Conjugate with a DNA Topoisomerase I Inhibitor DXd, Is Efficacious in Human Ovarian and Kidney Cancer Models. Mol. Cancer Ther..

[12] Huang Y., Clarke E., Garnett G.A.E., Lasalle M., Wong J., Wu A., Sagoe-Wagner A., Barbosa E., Lodaya K., Suarez C. In Vitro Assays to Predict ADC Hematological Toxicity: Contribution of Antibody, Linker, and Payload. http://www.zymeworks.com.

[13] Li S., Guo Y., Che J., Dai H., Dong X. (2025). Recent Advances in Peptide Linkers for Antibody-Drug Conjugates. J. Med. Chem..

[14] Zhang Y., Wang L., Cao X., Song R., Yin S., Cheng Z., Li W., Shen K., Zhao T., Xu J. (2024). Evaluation of Double Self-Immolative Linker-Based Antibody-Drug Conjugate FDA022-BB05 with Enhanced Therapeutic Potential. J. Med. Chem..

[15] Zhao C., Lu D., Gao J. (2025). Telisotuzumab Vedotin: The First-in-Class c-Met-Targeted Antibody-Drug Conjugate Granted FDA Accelerated Approval for Treatment of Non-Squamous Non-Small Cell Lung Cancer (NSCLC). Drug Discov. Ther..

[16] Wang D., Yin F., Li Z., Zhang Y., Shi C. (2025). Current Progress and Remaining Challenges of Peptide–Drug Conjugates (PDCs): Next Generation of Antibody-Drug Conjugates (ADCs)?. J. Nanobiotechnol..

[17] U.S. Department of Health and Human Services, National Institutes of Health, National Library of Medicine, National Center for Biotechnology Information Clinical Trials. https://www.clinicaltrials.gov/.

[18] U.S. Food and Drug Administration FDA-Approved Drugs. https://www.accessdata.fda.gov/scripts/cder/daf/index.cfm.

[19] Shastry M., Jacob S., Rugo H.S., Hamilton E. (2022). Antibody-Drug Conjugates Targeting TROP-2: Clinical Development in Metastatic Breast Cancer. Breast.

[20] Tsao L.C., Wang J.S., Ma X., Sodhi S., Ragusa J.V., Liu B., McBane J., Wang T., Wei J., Liu C.X. (2025). Effective Extracellular Payload Release and Immunomodulatory Interactions Govern the Therapeutic Effect of Trastuzumab Deruxtecan (T-DXd). Nat. Commun..

[21] Fu Z., Li S., Han S., Shi C., Zhang Y. (2022). Antibody Drug Conjugate: The “Biological Missile” for Targeted Cancer Therapy. Signal Transduct. Target. Ther..

[22] Blackhall F., Jao K., Greillier L., Cho B.C., Penkov K., Reguart N., Majem M., Nackaerts K., Syrigos K., Hansen K. (2021). Efficacy and Safety of Rovalpituzumab Tesirine Compared With Topotecan as Second-Line Therapy in DLL3-High SCLC: Results From the Phase 3 TAHOE Study. J. Thorac. Oncol..

[23] Stein E.M., Walter R.B., Erba H.P., Fathi A.T., Advani A.S., Lancet J.E., Ravandi F., Kovacsovics T., Deangelo D.J., Bixby D. (2018). A Phase 1 Trial of Vadastuximab Talirine as Monotherapy in Patients with CD33-Positive Acute Myeloid Leukemia. Blood.

[24] Wen L., Zhang Y., Sun C., Wang S.S., Gong Y., Jia C., Luo J. (2025). Fundamental Properties and Principal Areas of Focus in Antibody–Drug Conjugates Formulation Development. Antib. Ther..

[25] Jen E.Y., Ko C.W., Eun Lee J., Del Valle P.L., Aydanian A., Jewell C., Norsworthy K.J., Przepiorka D., Nie L., Liu J. (2018). Fda Approval: Gemtuzumab Ozogamicin for the Treatment of Adults with Newly Diagnosed Cd33-Positive Acute Myeloid Leukemia. Clin. Cancer Res..

[26] Ross P.L., Wolfe J.L. (2016). Physical and Chemical Stability of Antibody Drug Conjugates: Current Status. J. Pharm. Sci..

[27] Duerr C., Friess W. (2019). Antibody-Drug Conjugates- Stability and Formulation. Eur. J. Pharm. Biopharm..

[28] Li M., Zhao X., Yu C., Wang L. (2024). Antibody-Drug Conjugate Overview: A State-of-the-Art Manufacturing Process and Control Strategy. Pharm. Res..

[29] Álvarez-Palencia Jiménez R., Maze A., Bruckert F., Bensaid F., El-Kechai N., Weidenhaupt M. (2024). Evaluating Surfactant Effectiveness in Preventing Antibody Adsorption Directly on Medical Surfaces Using a Novel Device. Eur. J. Pharm. Biopharm..

[30] Weber J., Buske J., Mäder K., Garidel P., Diederichs T. (2023). Oxidation of Polysorbates—An Underestimated Degradation Pathway?. Int. J. Pharm. X.

[31] Zheng X., Sutton A.T., Yang R.S., Miller D.V., Pagels B., Rustandi R.R., Welch J., Payne A., Haverick M. (2023). Extensive Characterization of Polysorbate 80 Oxidative Degradation Under Stainless Steel Conditions. J. Pharm. Sci..

[32] Nguyen T.D., Bordeau B.M., Balthasar J.P. (2023). Mechanisms of ADC Toxicity and Strategies to Increase ADC Tolerability. Cancers.

[33] Balamkundu S., Liu C.F. (2023). Lysosomal-Cleavable Peptide Linkers in Antibody–Drug Conjugates. Biomedicines.

[34] Bhat R., Bhandary A., Karicheri V., Palakki G., Uday M. (2025). Antibody-Drug Conjugates: Advances and Applications in Targeted Cancer Therapies. Oral Sphere J. Dent. Health Sci..

[35] Buongervino S., Lane M.V., Garrigan E., Zhelev D.V., Dimitrov D.S., Bosse K.R. (2021). Antibody-Drug Conjugate Efficacy in Neuroblastoma: Role of Payload, Resistance Mechanisms, Target Density, and Antibody Internalization. Mol. Cancer Ther..

[36] Huang H., Zhou Y., Shang C., Zhang Y., Shen Y. (2025). A Novel Anti-HER2/EGFR Bispecific Antibody–Drug Conjugate Demonstrates Promising Antitumor Efficacy and Overcomes Resistance to HER2- or EGFR-Targeted ADCs. Investig. New Drugs.

[37] Probst P., Attinger-Toller I., Bertrand R., Stark R., Santimaria R., Schlereth B., Grabulovski D., Spycher P.R. (2025). Broadening the Therapeutic Window of ADCs Using Site-Specific Bioconjugation Showcased by an MMAE-Containing Peptide Linker in a CD79b-Targeting ADC. Mol. Cancer Ther..

[38] Liu J., Yang J., Sun Y., Gong J., Yue J., Pan Y., Sun M., Song R., Xiao X., Tazbirkova A. (2025). CLDN18.2–Targeting Antibody–Drug Conjugate IBI343 in Advanced Gastric or Gastroesophageal Junction Adenocarcinoma: A Phase 1 Trial. Nat. Med..

[39] Daver N.G., Montesinos P., DeAngelo D.J., Wang E.S., Papadantonakis N., Todisco E., Sweet K.L., Pemmaraju N., Lane A.A., Torres-Miñana L. (2024). Pivekimab Sunirine (IMGN632), a Novel CD123-Targeting Antibody–Drug Conjugate, in Relapsed or Refractory Acute Myeloid Leukaemia: A Phase 1/2 Study. Lancet Oncol..

[40] Chen B., Zheng X., Wu J., Chen G., Yu J., Xu Y., Wu W.K.K., Tse G.M.K., To K.F., Kang W. (2025). Antibody–Drug Conjugates in Cancer Therapy: Current Landscape, Challenges, and Future Directions. Mol. Cancer.

[41] Su D., Zhang D. (2021). Linker Design Impacts Antibody-Drug Conjugate Pharmacokinetics and Efficacy via Modulating the Stability and Payload Release Efficiency. Front. Pharmacol..

[42] Tan H.N., Morcillo M.A., Lopez J., Minchom A., Sharp A., Paschalis A., Silva-Fortes G., Raobaikady B., Banerji U. (2025). Treatment-Related Adverse Events of Antibody Drug-Conjugates in Clinical Trials. J. Hematol. Oncol..

[43] Andriollo P., di Mascio D., Jackson P.J.M., Hasan M.M., Pysz-Hosey I., Procopiou G., Fox K.R., Rahman K.M., Thurston D.E. (2025). A Novel DNA Sequence-Selective, Guanine Mono-Alkylating ADC Payload Suitable for Solid Tumour Treatment. RSC Med. Chem..

[44] Best R.L., LaPointe N.E., Azarenko O., Miller H., Genualdi C., Chih S., Shen B.Q., Jordan M.A., Wilson L., Feinstein S.C. (2021). Microtubule and Tubulin Binding and Regulation of Microtubule Dynamics by the Antibody Drug Conjugate (ADC) Payload, Monomethyl Auristatin E (MMAE): Mechanistic Insights into MMAE ADC Peripheral Neuropathy. Toxicol. Appl. Pharmacol..

[45] Lee G., Iwase T., Matsumoto S., Nabil A., Ebara M. (2023). Development of Apoptotic-Cell-Inspired Antibody–Drug Conjugate for Effective Immune Modulation. Int. J. Mol. Sci..

[46] Jafary B., Akbarzadeh-Khiavi M., Farzi-Khajeh H., Safary A., Adibkia K. (2025). EGFR-Targeting RNase A-Cetuximab Antibody-Drug Conjugate Induces ROS-Mediated Apoptosis to Overcome Drug Resistance in KRAS Mutant Cancer Cells. Sci. Rep..

[47] Lim H., Ma G., Jeong Y., Lee J.H., Lee J.H., Yang S.B., Choi J.U., Kim H.R., Park J. (2024). Tumor Targeting in Situ Aggregation of Nanoparticle-to-Nanoparticle Strategy to Simultaneously Induce Ferroptosis and Immunogenic Cell Death Using Complementary Heparin and Protamine Molecules. Nano Today.

[48] Rao M., Murali S., Amores D., Yu F., Tsourkas A. (2024). Exploring the Sensitivity of Antibody-Drug Conjugate Efficacy to the Selection of Payload, Antibody, and Cell Line. Bioconjug. Chem..

[49] Wang J., Lin S., Zhang J., Su J. (2025). A Meta-Analysis and Systematic Review of the First Trop-2-Targeting Antibody–Drug Conjugate (Sacituzumab Govitecan) in Treating Metastatic Breast Cancer. Eur. J. Clin. Pharmacol..

[50] Hafeez U., Parakh S., Gan H.K., Scott A.M. (2020). Antibody–drug Conjugates for Cancer Therapy. Molecules.

[51] Zhang S., Sun T., Wang X., Yan M., Liu Q., Tong Z., Yin Y., Yu G., Wang J., Su W. (2025). Efficacy and Safety of MRG002 Monotherapy in Treating Patients with Locally Advanced or Metastatic Breast Cancer with Low HER2 Expression: A Multi-Center, Non-Randomized, Open-Label Phase II Clinical Trial. Eur. J. Cancer.

[52] Mauricio D., Bellone S., Mutlu L., McNamara B., Manavella D.D., Demirkiran C., Verzosa M.S.Z., Buza N., Hui P., Hartwich T.M.P. (2023). Trastuzumab Deruxtecan (DS-8201a), a HER2-Targeting Antibody–Drug Conjugate with Topoisomerase I Inhibitor Payload, Shows Antitumor Activity in Uterine and Ovarian Carcinosarcoma with HER2/Neu Expression. Gynecol. Oncol..

[53] Amatore F., Ortonne N., Lopez M., Orlanducci F., Castellano R., Ingen-Housz-Oro S., de Croos A., Salvado C., Gorvel L., Goubard A. (2020). ICOS Is Widely Expressed in Cutaneous T-Cell Lymphoma, and Its Targeting Promotes Potent Killing of Malignant Cells. Blood Adv..

[54] Seki T., Yamada K., Ooba Y., Fujii T., Narita T., Nakayama A., Kitahara Y., Mendelsohn B.A., Matsuda Y., Okuzumi T. (2022). Biological Evaluation of Maytansinoid-Based Site-Specific Antibody-Drug Conjugate Produced by Fully Chemical Conjugation Approach: AJICAP^®^. Front. Biosci.-Landmark.

[55] Xue Q., Peng J., Dai W., Wu Q., Jiao J., Hu Y., Sha W., Yang Y., Yu W., Liu S. (2025). A Biparatopic HER2-Targeting ADC Constructed via Site-Specific Glycan Conjugation Exhibits Superior Stability, Safety, and Efficacy. RSC Chem. Biol..

[56] Lebar B., Zidar M., Mravljak J., Šink R., Žula A., Pajk S. (2024). Alternative Buffer Systems in Biopharmaceutical Formulations and Their Effect on Protein Stability. Acta Pharm..

[57] Garidel P., Blume A., Wagner M. (2015). Prediction of Colloidal Stability of High Concentration Protein Formulations. Pharm. Dev. Technol..

[58] Fu C., Zhang Z., Zhou S., Pritts W.A., Zhang Q. (2020). Assessing Localized Conformational Stability of Antibody-Drug Conjugate by Protein Conformation Assay. J. Pharm. Biomed. Anal..

[59] Geiger T., Clarke S. (1987). Deamidation, Isomerization, and Racemization at Asparaginyl and Aspartyl Residues in Peptides. Succinimide-Linked Reactions That Contribute to Protein Degradation. J. Biol. Chem..

[60] Cao M., Hussmann G.P., Tao Y., O’Connor E., Parthemore C., Zhang-Hulsey D., Liu D., Jiao Y., de Mel N., Prophet M. (2023). Atypical Asparagine Deamidation of NW Motif Significantly Attenuates the Biological Activities of an Antibody Drug Conjugate. Antibodies.

[61] Hamann P.R., Hinman L.M., Hollander I., Beyer C.F., Lindh D., Holcomb R., Hallett W., Tsou H.R., Upeslacis J., Shochat D. (2002). Gemtuzumab Ozogamicin, a Potent and Selective Anti-CD33 Antibody—Calicheamicin Conjugate for Treatment of Acute Myeloid Leukemia. Bioconjug. Chem..

[62] Glover Z.K., Wecksler A., Aryal B., Mehta S., Pegues M., Chan W., Lehtimaki M., Luo A., Sreedhara A., Rao V.A. (2022). Physicochemical and Biological Impact of Metal-Catalyzed Oxidation of IgG1 Monoclonal Antibodies and Antibody-Drug Conjugates via Reactive Oxygen Species. mAbs.

[63] Thallaj N. (2025). Analyzing Charge Variant Profiles of Monoclonal Antibodies Conjugated to Cytotoxic Agents. Int. J. Adv. Pharm. Sci. Res..

[64] Yang J., Tan H.Y., Yuan J., Huang Y., Rosenbaum A.I. (2025). Detailed Structural Elucidation of Antibody-Drug Conjugate Biotransformation Species Using High Resolution Multiple Reaction Monitoring Mass Spectrometry with Orthogonal Dissociation Methods. ACS Pharmacol. Transl. Sci..

[65] Akbarian M., Chen S.H. (2022). Instability Challenges and Stabilization Strategies of Pharmaceutical Proteins. Pharmaceutics.

[66] Ren S. (2023). Effects of Arginine in Therapeutic Protein Formulations: A Decade Review and Perspectives. Antib. Ther..

[67] D’Atri V., Imiołek M., Quinn C., Finny A., Lauber M., Fekete S., Guillarme D. (2024). Size Exclusion Chromatography of Biopharmaceutical Products: From Current Practices for Proteins to Emerging Trends for Viral Vectors, Nucleic Acids and Lipid Nanoparticles. J. Chromatogr. A.

[68] Vargas S.K., Eskafi A., Carter E., Ciaccio N. (2020). A Comparison of Background Membrane Imaging versus Flow Technologies for Subvisible Particle Analysis of Biologics. Int. J. Pharm..

[69] Leng C., Sun S., Lin W., Pavon J.A., Gennaro L., Gunawan R.C., Bu X., Yang T., Li S. (2024). Imaged Capillary Isoelectric Focusing Method Development for Charge Variants of High DAR ADCs. Anal. Chim. Acta.

[70] Yang F., Zhang J., Buettner A., Vosika E., Sadek M., Hao Z., Reusch D., Koenig M., Chan W., Bathke A. (2023). Mass Spectrometry-Based Multi-Attribute Method in Protein Therapeutics Product Quality Monitoring and Quality Control. mAbs.

[71] Ouyang J. (2013). Drug-to-Antibody Ratio (DAR) and Drug Load Distribution by Hydrophobic Interaction Chromatography and Reversed Phase High-Performance Liquid Chromatography. Methods Mol. Biol..

[72] Nguyen K.T.T., Zillen D., Lasorsa A., van der Wel P.C.A., Frijlink H.W., Hinrichs W.L.J. (2024). Combinations of Arginine and Pullulan Reveal the Selective Effect of Stabilization Mechanisms on Different Lyophilized Proteins. Int. J. Pharm..

[73] Johann F., Wöll S., Gieseler H. (2024). Evaluating the Potential of Cyclodextrins in Reducing Aggregation of Antibody–Drug Conjugates with Different Payloads. J. Pharm. Sci..

[74] Beckley N.S., Lazzareschi K.P., Chih H.W., Sharma V.K., Flores H.L. (2013). Investigation into Temperature-Induced Aggregation of an Antibody Drug Conjugate. Bioconjug. Chem..

[75] Sert F., Hız D., Gülmez M., Cankurtaran S.E., Kayalan C.I., Kurt H., Yüce M. (2022). Temperature and PH-Dependent Behaviors of MAb Drugs: A Case Study for Trastuzumab. Sci. Pharm..

[76] Buecheler J.W., Winzer M., Weber C., Gieseler H. (2019). Oxidation-Induced Destabilization of Model Antibody-Drug Conjugates. J. Pharm. Sci..

[77] Klair N., Kim M.T., Lee A., Xiao N.J., Patel A.R. (2021). Stress Temperature Studies in Small Scale Hastelloy^®^ Drug Substance Containers Lead to Increased Extent of and Increased Variability in Antibody-Drug Conjugate and Monoclonal Antibody Aggregation: Evidence for Novel Oxidation-Induced Crosslinking in Monoclonal Antibodies. J. Pharm. Sci..

[78] Friese O.V., Smith J.N., Brown P.W., Rouse J.C. (2018). Practical Approaches for Overcoming Challenges in Heightened Characterization of Antibody-Drug Conjugates with New Methodologies and Ultrahigh-Resolution Mass Spectrometry. mAbs.

[79] Buecheler J.W., Winzer M., Tonillo J., Weber C., Gieseler H. (2018). Impact of Payload Hydrophobicity on the Stability of Antibody-Drug Conjugates. Mol. Pharm..

[80] Hobson A.D., Zhu H., Qiu W., Judge R.A., Longenecker K. (2024). Minimising the Payload Solvent Exposed Hydrophobic Surface Area Optimises the Antibody-Drug Conjugate Properties. RSC Med. Chem..

[81] Junutula J.R., Raab H., Clark S., Bhakta S., Leipold D.D., Weir S., Chen Y., Simpson M., Tsai S.P., Dennis M.S. (2008). Site-Specific Conjugation of a Cytotoxic Drug to an Antibody Improves the Therapeutic Index. Nat. Biotechnol..

[82] Tang S.C., Wynn C., Le T., McCandless M., Zhang Y., Patel R., Maihle N., Hillegass W. (2025). Influence of Antibody–Drug Conjugate Cleavability, Drug-to-Antibody Ratio, and Free Payload Concentration on Systemic Toxicities: A Systematic Review and Meta-Analysis. Cancer Metastasis Rev..

[83] Song W., Yin L., Ren J., Jiang L., Huang Z.A., Fan Y., Tao R., Duan T., Su Z., Cao Y. (2025). Simultaneous Analysis of the Drug-to-Antibody Ratio, Free-Drug-Related Impurities, and Purity of Antibody-Drug Conjugates Based on Size Exclusion Chromatography. Anal. Chem..

[84] Källsten M., Hartmann R., Kovac L., Lehmann F., Lind S.B., Bergquist J. (2020). Investigating the Impact of Sample Preparation on Mass Spectrometry-Based Drug-to-Antibody Ratio Determination for Cysteine-and Lysine-Linked Antibody–Drug Conjugates. Antibodies.

[85] Lofgren K.A., Sreekumar S., Jenkins E.C., Ernzen K.J., Kenny P.A. (2021). Anti-Tumor Efficacy of an MMAE-Conjugated Antibody Targeting Cell Surface TACE/ADAM17-Cleaved Amphiregulin in Breast Cancer. Antib. Ther..

[86] Wang Y., Liu L., Fan S., Xiao D., Xie F., Li W., Zhong W., Zhou X. (2020). Antibody-Drug Conjugate Using Ionized CYS-Linker-Mmae as the Potent Payload Shows Optimal Therapeutic Safety. Cancers.

[87] Wan G., Yang L., Wang Q., Xu G. (2025). T-DM1 with Concurrent Radiotherapy in HER2-Positive Breast Cancer: Preclinical Evaluation and Mechanisms, Prediction, and Exploration of Adverse Effects. Discov. Oncol..

[88] Xiao D., Zhao L., Xie F., Fan S., Liu L., Li W., Cao R., Li S., Zhong W., Zhou X. (2021). A Bifunctional Molecule-Based Strategy for the Development of Theranostic Antibody-Drug Conjugate. Theranostics.

[89] Su A., Luo Y., Zhang C., Duan H. (2025). Linker-GPT: Design of Antibody-Drug Conjugates Linkers with Molecular Generators and Reinforcement Learning. Sci. Rep..

[90] Sasso J.M., Tenchov R., Bird R., Iyer K.A., Ralhan K., Rodriguez Y., Zhou Q.A. (2023). The Evolving Landscape of Antibody-Drug Conjugates: In Depth Analysis of Recent Research Progress. Bioconjug. Chem..

[91] Wei C., Zhang G., Clark T., Barletta F., Tumey L.N., Rago B., Hansel S., Han X. (2016). Where Did the Linker-Payload Go? A Quantitative Investigation on the Destination of the Released Linker-Payload from an Antibody-Drug Conjugate with a Maleimide Linker in Plasma. Anal. Chem..

[92] Ji J.A., Liu J., Wang Y.J. (2025). Formulation Development for Antibody–Drug Conjugates. Antibody-Drug Conjugates.

[93] Bargh J.D., Isidro-Llobet A., Parker J.S., Spring D.R. (2019). Cleavable Linkers in Antibody-Drug Conjugates. Chem. Soc. Rev..

[94] Jain N., Smith S.W., Ghone S., Tomczuk B. (2015). Current ADC Linker Chemistry. Pharm. Res..

[95] Giese M., Davis P.D., Woodman R.H., Hermanson G., Pokora A., Vermillion M. (2021). Linker Architectures as Steric Auxiliaries for Altering Enzyme-Mediated Payload Release from Bioconjugates. Bioconjug. Chem..

[96] Bargh J.D., Walsh S.J., Ashman N., Isidro-Llobet A., Carroll J.S., Spring D.R. (2021). A Dual-Enzyme Cleavable Linker for Antibody-Drug Conjugates. Chem. Commun..

[97] Su Z., Xiao D., Xie F., Liu L., Wang Y., Fan S., Zhou X., Li S. (2021). Antibody–Drug Conjugates: Recent Advances in Linker Chemistry. Acta Pharm. Sin. B.

[98] Brülisauer L., Gauthier M.A., Leroux J.C. (2014). Disulfide-Containing Parenteral Delivery Systems and Their Redox-Biological Fate. J. Control. Release.

[99] Nagy P. (2013). Kinetics and Mechanisms of Thiol-Disulfide Exchange Covering Direct Substitution and Thiol Oxidation-Mediated Pathways. Antioxid. Redox Signal..

[100] Carneiro A., Santana L., Matos M.J. (2023). Oxidation-Labile Linkers for Controlled Drug Delivery. Bioorg. Med. Chem. Lett..

[101] Kozuch B., Weber J., Buske J., Mäder K., Garidel P., Diederichs T. (2023). Comparative Stability Study of Polysorbate 20 and Polysorbate 80 Related to Oxidative Degradation. Pharmaceutics.

[102] Guo J., Kumar S., Prashad A., Starkey J., Singh S.K. (2014). Assessment of Physical Stability of an Antibody Drug Conjugate by Higher Order Structure Analysis: Impact of Thiol-Maleimide Chemistry. Pharm. Res..

[103] Tumey L.N., Charati M., He T., Sousa E., Ma D., Han X., Clark T., Casavant J., Loganzo F., Barletta F. (2014). Mild Method for Succinimide Hydrolysis on ADCs: Impact on ADC Potency, Stability, Exposure, and Efficacy. Bioconjug. Chem..

[104] Lyon R.P., Setter J.R., Bovee T.D., Doronina S.O., Hunter J.H., Anderson M.E., Balasubramanian C.L., Duniho S.M., Leiske C.I., Li F. (2014). Self-Hydrolyzing Maleimides Improve the Stability and Pharmacological Properties of Antibody-Drug Conjugates. Nat. Biotechnol..

[105] Hernandez-Barry H., dela Cruz-Chuh J., Kajihara K.K., Asundi J., Vandlen R., Zhang D., Hazenbos W.L.W., Pillow T., Liu Y., Wu C. (2025). Mechanistic Characterization of the Potency of THIOMAB Antibody-Drug Conjugates Targeting Staphylococcus Aureus and ETbR-Expressing Tumor Cells Using Quantitative LC-MS/MS Analysis of Intracellular Drug Accumulation. Bioconjug. Chem..

[106] Tedeschini T., Campara B., Grigoletto A., Bellini M., Salvalaio M., Matsuno Y., Suzuki A., Yoshioka H., Pasut G. (2021). Polyethylene Glycol-Based Linkers as Hydrophilicity Reservoir for Antibody-Drug Conjugates. J. Control. Release.

[107] Conilh L., Fournet G., Fourmaux E., Murcia A., Matera E.L., Joseph B., Dumontet C., Viricel W. (2021). Exatecan Antibody Drug Conjugates Based on a Hydrophilic Polysarcosine Drug-Linker Platform. Pharmaceuticals.

[108] Zambra M., Ranđelović I., Talarico F., Borbély A., Svajda L., Tóvári J., Mező G., Bodero L., Colombo S., Arrigoni F. (2023). Optimizing the Enzymatic Release of MMAE from IsoDGR-Based Small Molecule Drug Conjugate by Incorporation of a GPLG-PABC Enzymatically Cleavable Linker. Front. Pharmacol..

[109] Fishkin N., Maloney E.K., Chari R.V.J., Singh R. (2011). A Novel Pathway for Maytansinoid Release from Thioether Linked Antibody-Drug Conjugates (ADCs) under Oxidative Conditions. Chem. Commun..

[110] Zheng K., Chen Y., Wang J., Zheng L., Hutchinson M., Persson J., Ji J. (2019). Characterization of Ring-Opening Reaction of Succinimide Linkers in ADCs. J. Pharm. Sci..

[111] Huang L., Qin G., Gong C., Sun Y., Yang H., Lv C., Liu C., Jiang L., Yuan J., Hu M. (2025). Site-Specific Ligase-Dependent Conjugation with Ring-Opening Linker Improves Safety and Stability of HER2-Targeting ADCs. Nat. Commun..

[112] Long J., Shao T., Wang Y., Chen T., Chen Y., Chen Y.L., Wang Q., Yu X., Yu J., He K. (2025). PEGylation of Dipeptide Linker Improves Therapeutic Index and Pharmacokinetics of Antibody-Drug Conjugates. Bioconjug. Chem..

[113] Cao A., Chen J.Y. (2025). Generating Novel Linker Structures in Antibody-Drug Conjugates with Diffusion Models. Artificial Intelligence in Medicine, Proceedings of the AIME 2025, Pavia, Italy, 23–26 June 2025.

[114] Qiu D., Huang Y., Chennamsetty N., Miller S.A., Hay M. (2021). Characterizing and Understanding the Formation of Cysteine Conjugates and Other By-Products in a Random, Lysine-Linked Antibody Drug Conjugate. mAbs.

[115] Luo C., Ren A., Jin Z., Zhang J., Shi W., Zeng Y., Liu Z., Lu M., Hou Y., Tang F. (2024). Design and Synthesis of Novel Site-Specific Antibody-Drug Conjugates That Target TROP2. Bioorg. Med. Chem..

[116] Fujii T., Matsuda Y., Seki T., Shikida N., Iwai Y., Ooba Y., Takahashi K., Isokawa M., Kawaguchi S., Hatada N. (2023). AJICAP Second Generation: Improved Chemical Site-Specific Conjugation Technology for Antibody-Drug Conjugate Production. Bioconjug. Chem..

[117] Kumar D., Trivedi N. (2026). Advances in LC–MS Strategies for Comprehensive Characterization and Quantification of Antibody-Drug Conjugates in Preclinical and Clinical Settings. J. Chromatogr. A.

[118] Kumar A., Mao S., Dimasi N., Gao C. (2020). Design and Validation of Linkers for Site-Specific Preparation of Antibody–Drug Conjugates Carrying Multiple Drug Copies per Cysteine Conjugation Site. Int. J. Mol. Sci..

[119] Hengel S.M., Topletz-Erickson A.R., Kadry H., Alley S.C. (2024). A Modelling Approach to Compare ADC Deconjugation and Systemic Elimination Rates of Individual Drug-Load Species Using Native ADC LC-MS Data from Human Plasma. Xenobiotica.

[120] Moquist P.N., Eng-Duncan N.M.L., Bovee T.D., Snead K., Bou L., Zhang X., Blackburn B., Wright J., Simmons J.K., Neff-LaFord H.D. (2025). Auristatin S: An Auristatin Payload with Improved Tolerability and Modified Bystander Activity. ACS Med. Chem. Lett..

[121] Zou J., Kinosada H., Takayanagi S.I., Ishii T., Amano T., Nihira K., Kanie S., Adachi M., Tahara H., Sakoda T. (2025). KK2845, a PBD Dimer-Containing Antibody-Drug Conjugate Targeting TIM-3-Expressing AML. Leukemia.

[122] Wiedemeyer W.R., Gavrilyuk J., Schammel A., Zhao X., Sarvaiya H., Pysz M., Gu C., You M., Isse K., Sullivan T. (2022). ABBV-011, A Novel, Calicheamicin-Based Antibody–Drug Conjugate, Targets SEZ6 to Eradicate Small Cell Lung Cancer Tumors. Mol. Cancer Ther..

[123] Cheng Y., Yuan X., Tian Q., Huang X., Chen Y., Pu Y., Long H., Xu M., Ji Y., Xie J. (2022). Preclinical Profiles of SKB264, a Novel Anti-TROP2 Antibody Conjugated to Topoisomerase Inhibitor, Demonstrated Promising Antitumor Efficacy Compared to IMMU-132. Front. Oncol..

[124] Sokka I.K., Imlimthan S., Sarparanta M., Maaheimo H., Johansson M.P., Ekholm F.S. (2021). Halogenation at the Phenylalanine Residue of Monomethyl Auristatin F Leads to a Favorable Cis/Trans Equilibrium and Retained Cytotoxicity. Mol. Pharm..

[125] Stanley-Smith A.E., Coates N., Fox J.C., Delfino F., Degrado S., Kunz A., Mao S., Zhao F., Garforth B., Hold A. (2025). Mutasynthesis of C17-and C21-Substituted Ansamitocins for Use as ADC Payloads. ACS Omega.

[126] Sun W., Weng W., Shi J., Ma B., DeMarco K.D., Gui F., Jin R., Ruscetti M., Jia L., Hu W. (2025). SPARC: A Multipayload ADC Architecture for Programmable Drug Combinations. Bioconjug. Chem..

[127] Thomas J.D., Yurkovetskiy A.V., Yin M., Bodyak N.D., Gumerov D.R., Tang S., Kelleher E., Jones B.D., Protopopova M., Qin L.L. (2022). Discovery of Novel Polyamide-Pyrrolobenzodiazepine Hybrids for Antibody-Drug Conjugates. Bioorg. Med. Chem. Lett..

[128] Luo S., Bulos J., Uroza R., Zhao Y., Pan X., Su Y., Qiu H., Olagunju B., Wang W., Liu D. (2025). Insights into Photo Degradation and Stabilization Strategies of Antibody–Drug Conjugates with Camptothecin Payloads. Pharmaceutics.

[129] Thiess C.O., Mäder K., Mahler H.-C., Wöll S. (2025). Payload-Dependent Photostability of Antibody-Drug Conjugates. Eur. J. Pharm. Biopharm..

[130] Satomaa T., Pynnönen H., Vilkman A., Kotiranta T., Pitkänen V., Heiskanen A., Herpers B., Price L.S., Helin J., Saarinen J. (2018). Hydrophilic Auristatin Glycoside Payload Enables Improved Antibody-Drug Conjugate Efficacy and Biocompatibility. Antibodies.

[131] Johann F., Wöll S., Gieseler H. (2024). “Negative” Impact: The Role of Payload Charge in the Physicochemical Stability of Auristatin Antibody–Drug Conjugates. J. Pharm. Sci..

[132] Thorsteinson N., Gunn J.R., Kelly K., Long W., Labute P. (2021). Structure-Based Charge Calculations for Predicting Isoelectric Point, Viscosity, Clearance, and Profiling Antibody Therapeutics. mAbs.

[133] Widdison W., Wilhelm S., Veale K., Costoplus J., Jones G., Audette C., Leece B., Bartle L., Kovtun Y., Chari R. (2015). Metabolites of Antibody-Maytansinoid Conjugates: Characteristics and in Vitro Potencies. Mol. Pharm..

[134] Qi M., Zhu C., Chen Y., Wang C., Ye X., Li S., Cheng Z., Jiang H., Du Z. (2024). Site-Specific Stability Evaluation of Antibody-Drug Conjugate in Serum Using a Validated Liquid Chromatography-Mass Spectrometry Method. J. Proteome Res..

[135] Cordova J.C., Sun S., Bos J., Thirumalairajan S., Ghone S., Hirai M., Busse R.A., der Hardt J.S.V., Schwartz I., Zhou J. (2021). Development of a Single-Step Antibody–Drug Conjugate Purification Process with Membrane Chromatography. J. Clin. Med..

[136] Hines A.R., Edgeworth M., Devine P.W.A., Shepherd S., Chatterton N., Turner C., Lilley K.S., Chen X., Bond N.J. (2023). Multi-Attribute Monitoring Method for Process Development of Engineered Antibody for Site-Specific Conjugation. J. Am. Soc. Mass Spectrom..

[137] Bai C., Reid E.E., Wilhelm A., Shizuka M., Maloney E.K., Laleau R., Harvey L., Archer K.E., Vitharana D., Adams S. (2020). Site-Specific Conjugation of the Indolinobenzodiazepine DGN549 to Antibodies Affords Antibody-Drug Conjugates with an Improved Therapeutic Index as Compared with Lysine Conjugation. Bioconjug. Chem..

[138] Wang Z., Li H., Gou L., Li W., Wang Y. (2023). Antibody–Drug Conjugates: Recent Advances in Payloads. Acta Pharm. Sin. B.

[139] Fujii T., Reiling C., Quinn C., Kliman M., Mendelsohn B.A., Matsuda Y. (2021). Physical Characteristics Comparison between Maytansinoid-Based and Auristatin-Based Antibody-Drug Conjugates. Explor. Target. Antitumor Ther..

[140] Scott C., Ojha S., Hough S., Stanton D., Abbott J., Studer M., Song-Young K. Antibody-Drug Conjugates Continuing Evolution in Manufacturing Strategy. https://www.bioprocessintl.com/emerging-therapeutics-manufacturing/ebook-antibody-drug-conjugates-continuing-evolution-in-manufacturing-strategy.

[141] Yamazoe S., Cheng Q., Kotapati S., Rangan V.S., Sung M.C., Deshpande M., Jashnani A., Qiang C., Smith M.J., Pan C. (2025). The Impact of Conjugation Mode and Site on Tubulysin Antibody-Drug-Conjugate Efficacy and Stability. ChemistryOpen.

[142] Matsuda Y., Seki T., Yamada K., Ooba Y., Takahashi K., Fujii T., Kawaguchi S., Narita T., Nakayama A., Kitahara Y. (2021). Chemical Site-Specific Conjugation Platform to Improve the Pharmacokinetics and Therapeutic Index of Antibody-Drug Conjugates. Mol. Pharm..

[143] Karunaratne S.P., Moussa E.M., Mills B.J., Weis D.D. (2024). Understanding the Effects of Site-Specific Light Chain Conjugation on Antibody Structure Using Hydrogen Exchange-Mass Spectrometry (HX-MS). J. Pharm. Sci..

[144] Watanabe T., Iwai Y., Stofleth J.T., Fujii T., Seki T., Matsuda Y. (2025). Homogeneous Dual-Payload Antibody-Drug Conjugates Produced by Combined Distinct Conjugation Strategies. ACS Med. Chem. Lett..

[145] Laber J.R., Laue T.M., Filoti D.I. (2022). Use of Debye-Hückel-Henry Charge Measurements in Early Antibody Development Elucidates Effects of Non-Specific Association. Antib. Ther..

[146] Ingavat N., Dzulkiflie N., Liew J.M., Wang X., Leong E., Loh H.P., Ng S.K., Yang Y., Zhang W. (2024). Investigation on Environmental Factors Contributing to Bispecific Antibody Stability and the Reversal of Self-Associated Aggregates. Bioresour. Bioprocess..

[147] Karunnanithy V., Abdul Rahman N.H.B., Abdullah N.A.H., Fauzi M.B., Lokanathan Y., Min Hwei A.N., Maarof M. (2024). Effectiveness of Lyoprotectants in Protein Stabilization During Lyophilization. Pharmaceutics.

[148] Svilenov H.L., Kulakova A., Zalar M., Golovanov A.P., Harris P., Winter G. (2020). Orthogonal Techniques to Study the Effect of PH, Sucrose, and Arginine Salts on Monoclonal Antibody Physical Stability and Aggregation During Long-Term Storage. J. Pharm. Sci..

[149] Cheng Y., Duong H.T.T., Hu Q., Shameem M., Tang X.C. (2025). Practical Advice in the Development of a Lyophilized Protein Drug Product. Antib. Ther..

[150] Haeuser C., Goldbach P., Huwyler J., Friess W., Allmendinger A. (2020). Impact of Dextran on Thermal Properties, Product Quality Attributes, and Monoclonal Antibody Stability in Freeze-Dried Formulations. Eur. J. Pharm. Biopharm..

[151] Saurabh S., Kalonia C., Li Z., Hollowell P., Waigh T., Li P., Webster J., Seddon J.M., Lu J.R., Bresme F. (2022). Understanding the Stabilizing Effect of Histidine on MAb Aggregation: A Molecular Dynamics Study. Mol. Pharm..

[152] Valliere-Douglass J.F., Lewis P., Salas-Solano O., Jiang S. (2015). Solid-State MAbs and ADCs Subjected to Heat-Stress Stability Conditions Can Be Covalently Modified with Buffer and Excipient Molecules. J. Pharm. Sci..

[153] Schuster J., Mahler H.C., Joerg S., Kamuju V., Huwyler J., Mathaes R. (2021). Stability of Monoclonal Antibodies after Simulated Subcutaneous Administration. J. Pharm. Sci..

[154] Ebrahimi S.B., Hong X., Ludlow J., Doucet D., Thirumangalathu R. (2023). Studying Intermolecular Interactions in an Antibody-Drug Conjugate Through Chemical Screening and Computational Modeling. J. Pharm. Sci..

[155] Zhou S., Schöneich C., Singh S.K. (2011). Biologics Formulation Factors Affecting Metal Leachables from Stainless Steel. AAPS PharmSciTech.

[156] Pardeshi S.R., Deshmukh N.S., Telange D.R., Nangare S.N., Sonar Y.Y., Lakade S.H., Harde M.T., Pardeshi C.V., Gholap A., Deshmukh P.K. (2023). Process Development and Quality Attributes for the Freeze-Drying Process in Pharmaceuticals, Biopharmaceuticals and Nanomedicine Delivery: A State-of-the-Art Review. Futur. J. Pharm. Sci..

[157] Zarzar J., Khan T., Bhagawati M., Weiche B., Sydow-Andersen J., Alavattam S. (2023). High Concentration Formulation Developability Approaches and Considerations. mAbs.

[158] Lai P.K., Gallegos A., Mody N., Sathish H.A., Trout B.L. (2022). Machine Learning Prediction of Antibody Aggregation and Viscosity for High Concentration Formulation Development of Protein Therapeutics. mAbs.

[159] Dash R., Rathore A.S. (2021). Freeze Thaw and Lyophilization Induced Alteration in MAb Therapeutics: Trastuzumab as a Case Study. J. Pharm. Biomed. Anal..

[160] Lyon R.P., Bovee T.D., Doronina S.O., Burke P.J., Hunter J.H., Neff-Laford H.D., Jonas M., Anderson M.E., Setter J.R., Senter P.D. (2015). Reducing Hydrophobicity of Homogeneous Antibody-Drug Conjugates Improves Pharmacokinetics and Therapeutic Index. Nat. Biotechnol..

[161] Hamblett K.J., Senter P.D., Chace D.F., Sun M.M.C., Lenox J., Cerveny C.G., Kissler K.M., Bernhardt S.X., Kopcha A.K., Zabinski R.F. (2004). Effects of Drug Loading on the Antitumor Activity of a Monoclonal Antibody Drug Conjugate. Clin. Cancer Res..

[162] U.S. Department of Health and Human Services, Food and Drug Administration, Center for Drug Evaluation and Research, Center for Biologics Evaluation and Research (2003). Guidance for Industry(R2) Stability Testing of New Drug Substances and Products.

[163] Liu K., Li M., Li Y., Li Y., Chen Z., Tang Y., Yang M., Deng G., Liu H. (2024). A Review of the Clinical Efficacy of FDA-Approved Antibody-drug Conjugates in Human Cancers. Mol. Cancer.

[164] Maecker H., Jonnalagadda V., Bhakta S., Jammalamadaka V., Junutula J.R. (2023). Exploration of the Antibody–Drug Conjugate Clinical Landscape. mAbs.

[165] Mckertish C.M., Kayser V. (2021). Advances and Limitations of Antibody Drug Conjugates for Cancer. Biomedicines.

[166] Manning M.C., Holcomb R.E., Payne R.W., Stillahn J.M., Connolly B.D., Katayama D.S., Liu H., Matsuura J.E., Murphy B.M., Henry C.S. (2024). Stability of Protein Pharmaceuticals: Recent Advances. Pharm. Res..

[167] Li J., Wang H., Wang L., Yu D., Zhang X. (2024). Stabilization Effects of Saccharides in Protein Formulations: A Review of Sucrose, Trehalose, Cyclodextrins and Dextrans. Eur. J. Pharm. Sci..

[168] Poudel Y.B., Chowdari N.S., Cheng H., Iwuagwu C.I., King H.D., Kotapati S., Passmore D., Rampulla R., Mathur A., Vite G. (2020). Chemical Modification of Linkers Provides Stable Linker-Payloads for the Generation of Antibody-Drug Conjugates. ACS Med. Chem. Lett..

[169] Matsuda Y., Chang J.R., Mendelsohn B.A. (2025). Advanced Antibody–Drug Conjugates Design: Innovation in Linker Chemistry and Site-Specific Conjugation Technologies. ChemBioChem.

[170] Andris S., Hubbuch J. (2020). Modeling of Hydrophobic Interaction Chromatography for the Separation of Antibody-Drug Conjugates and Its Application towards Quality by Design. J. Biotechnol..

[171] Matsuda Y., Leung M., Okuzumi T., Mendelsohn B. (2020). A Purification Strategy Utilizing Hydrophobic Interaction Chromatography to Obtain Homogeneous Species from a Site-Specific Antibody Drug Conjugate Produced by Ajicaptm First Generation. Antibodies.

[172] Zhu X.Y., Li Q.X., Kong Y., Huang K.K., Wang G., Wang Y.J., Lu J., Hua G.Q., Wu Y.L., Ying T.L. (2024). A Novel Human Single-Domain Antibody-Drug Conjugate CEACAM5 Exhibits Potent in Vitro and in Vivo Activity. Acta Pharmacol. Sin..

[173] Gandhi A.V., Arlotta K.J., Chen H.N., Owen S.C., Carpenter J.F. (2018). Biophysical Properties and Heating-Induced Aggregation of Lysine-Conjugated Antibody-Drug Conjugates. J. Pharm. Sci..

[174] Ling J., Du Y., Wuelfing W.P., Buist N., Krishnamachari Y., Xi H., Templeton A.C., Su Y. (2025). Molecular Mechanisms for Stabilizing Biologics in the Solid State. J. Pharm. Sci..

[175] Wagh A., Song H., Zeng M., Tao L., Das T.K. (2018). Challenges and New Frontiers in Analytical Characterization of Antibody-Drug Conjugates. mAbs.

[176] Li W., Prabakaran P., Chen W., Zhu Z., Feng Y., Dimitrov D.S. (2016). Antibody Aggregation: Insights from Sequence and Structure. Antibodies.

[177] Aoyama M., Tada M., Yokoo H., Demizu Y., Ishii-Watabe A. (2022). Fcγ Receptor-Dependent Internalization and Off-Target Cytotoxicity of Antibody-Drug Conjugate Aggregates. Pharm. Res..

[178] U.S. Department of Health and Human Services, Food and Drug Administration, Center for Drug Evaluation and Research, Center for Biologics Evaluation and Research (2021). Q12 Technical and Regulatory Considerations for Pharmaceutical Product Lifecycle Management Guidance for Industry.

[179] U.S. Department of Health and Human Services, Food and Drug Administration, Center for Drug Evaluation and Research, Center for Biologics Evaluation and Research (2022). Comparability Protocols for Postapproval Changes to the Chemistry, Manufacturing, and Controls Information in an NDA, ANDA, or BLA Guidance for Industry.

[180] U.S. Department of Health and Human Services, Food and Drug Administration, Center for Drug Evaluation and Research, Center for Biologics Evaluation and Research (2021). Chemistry, Manufacturing, and Controls Changes to an Approved Application: Certain Biological Products; Guidance for Industry.

[181] U.S. Department of Health and Human Services, Food and Drug Administration, Center for Drug Evaluation and Research, Center for Biologics Evaluation and Research (2004). Guidance for Industry Evaluation of Stability Data.

[182] U.S. Department of Health and Human Services, Food and Drug Administration, Center for Drug Evaluation and Research, Center for Biologics Evaluation and Research (2005). Guidance for Industry Q5E Comparability of Biotechnological/Biological Products Subject to Changes in Their Manufacturing Process.

[183] Luo S., McSweeney K.M., Wang T., Bacot S.M., Feldman G.M., Zhang B. (2020). Defining the Right Diluent for Intravenous Infusion of Therapeutic Antibodies. mAbs.

[184] Chen Y., Mutukuri T.T., Wilson N.E., Zhou Q. (2021). Pharmaceutical Protein Solids: Drying Technology, Solid-State Characterization and Stability. Adv. Drug Deliv. Rev..

[185] Cho E., Mayhugh B.M., Srinivasan J.M., Sacha G.A., Nail S.L., Topp E.M. (2021). Stability of Antibody Drug Conjugate Formulations Evaluated Using Solid-State Hydrogen-Deuterium Exchange Mass Spectrometry. J. Pharm. Sci..

[186] Zäh M., Brandenbusch C., Groël S., Winter G., Sadowski G. (2025). Water Activity as an Indicator for Antibody Storage Stability in Lyophilized Formulations. Mol. Pharm..

[187] Seifert I., Friess W. (2021). The Effect of Residual Moisture on a Monoclonal Antibody Stability in L-Arginine Based Lyophilisates. Eur. J. Pharm. Biopharm..

[188] Vallaster B., Engelsing F., Grohganz H. (2024). Influence of Water and Trehalose on α- and β-Relaxation of Freeze-Dried Lysozyme Formulations. Eur. J. Pharm. Biopharm..

[189] Ohtake S., Shalaev E. (2013). Effect of Water on the Chemical Stability of Amorphous Pharmaceuticals: I. Small Molecules. J. Pharm. Sci..

[190] Koshari S.H.S., Ross J.L., Nayak P.K., Zarraga I.E., Rajagopal K., Wagner N.J., Lenhoff A.M. (2017). Characterization of Protein-Excipient Microheterogeneity in Biopharmaceutical Solid-State Formulations by Confocal Fluorescence Microscopy. Mol. Pharm..

[191] Drogoń A., Skotnicki M., Skotnicka A., Pyda M. (2020). Physical Ageing of Amorphous Indapamide Characterised by Differential Scanning Calorimetry. Pharmaceutics.

[192] Clavaud M., Roggo Y., Dégardin K., Sacré P.Y., Hubert P., Ziemons E. (2016). Moisture Content Determination in an Antibody-Drug Conjugate Freeze-Dried Medicine by near-Infrared Spectroscopy: A Case Study for Release Testing. J. Pharm. Biomed. Anal..

[193] Gregson S.J., Barrett A.M., Patel N.V., Kang G.D., Schiavone D., Sult E., Barry C.S., Vijayakrishnan B., Adams L.R., Masterson L.A. (2019). Synthesis and Evaluation of Pyrrolobenzodiazepine Dimer Antibody-Drug Conjugates with Dual β-Glucuronide and Dipeptide Triggers. Eur. J. Med. Chem..

[194] Rubahamya B., Dong S., Thurber G.M. (2024). Clinical Translation of Antibody Drug Conjugate Dosing in Solid Tumors from Preclinical Mouse Data. Sci. Adv..

[195] Campbell G.A., Barrett S.E., Thakral S., Lowinger M., Faassen F., Vander Kamp K.A., Wagner D., Bargmann-Leyder N., Badawy S., Gaebele T. (2025). A Pharmaceutical Industry Survey Results on the Cycle Time, Drivers and Enablers, for Key Deliverables in the Early Phase of Drug Product Development from Dose Limiting Toxicity to First Subject First Dose: The Survey Conducted by the IQ Cycle Time Benchmarking Working Group. J. Drug Deliv. Sci. Technol..

[196] Li Y., Gu C., Gruenhagen J., Zhang K., Yehl P., Chetwyn N.P., Medley C.D. (2015). A Size Exclusion-Reversed Phase Two Dimensional-Liquid Chromatography Methodology for Stability and Small Molecule Related Species in Antibody Drug Conjugates. J. Chromatogr. A.

[197] Stoll D., Danforth J., Zhang K., Beck A. (2016). Characterization of Therapeutic Antibodies and Related Products by Two-Dimensional Liquid Chromatography Coupled with UV Absorbance and Mass Spectrometric Detection. J. Chromatogr. B Analyt. Technol. Biomed. Life Sci..

[198] Said N., Gahoual R., Kuhn L., Beck A., François Y.N., Leize-Wagner E. (2016). Structural Characterization of Antibody Drug Conjugate by a Combination of Intact, Middle-up and Bottom-up Techniques Using Sheathless Capillary Electrophoresis—Tandem Mass Spectrometry as NanoESI Infusion Platform and Separation Method. Anal. Chim. Acta.

[199] Bobály B., D’Atri V., Beck A., Guillarme D., Fekete S. (2017). Analysis of Recombinant Monoclonal Antibodies in Hydrophilic Interaction Chromatography: A Generic Method Development Approach. J. Pharm. Biomed. Anal..

[200] Carrillo R.J., Semple A. (2024). DSC Derived (Ea & ΔG) Energetics and Aggregation Predictions for MAbs. J. Pharm. Sci..

[201] Carvalho S.B., Gomes R.A., Pfenninger A., Fischer M., Strotbek M., Isidro I.A., Tugçu N., Gomes-Alves P. (2022). Multi Attribute Method Implementation Using a High Resolution Mass Spectrometry Platform: From Sample Preparation to Batch Analysis. PLoS ONE.

[202] Rogstad S., Yan H., Wang X., Powers D., Brorson K., Damdinsuren B., Lee S. (2019). Multi-Attribute Method for Quality Control of Therapeutic Proteins. Anal. Chem..

[203] Gervais A., Dirksen E.H.C., Pohl T., Bechtold-Peters K., Burkitt W., D’Alessio V., Greven S., Lennard A., Li X., Lössner C. (2023). Compliance and Regulatory Considerations for the Implementation of the Multi-Attribute-Method by Mass Spectrometry in a Quality Control Laboratory. Eur. J. Pharm. Biopharm..

[204] Rogstad S., Namuswe F. Quality Considerations for the Multi-Attribute Method (MAM) for Therapeutic Proteins. https://www.fda.gov/science-research/fda-grand-rounds/quality-considerations-multi-attribute-method-mam-therapeutic-proteins-10132022#event-materials.

[205] D’Atri V., Pell R., Clarke A., Guillarme D., Fekete S. (2019). Is Hydrophobic Interaction Chromatography the Most Suitable Technique to Characterize Site-Specific Antibody-Drug Conjugates?. J. Chromatogr. A.

[206] Wakankar A., Chen Y., Gokarn Y., Jacobson F.S. (2011). Analytical Methods for Physicochemical Characterization of Antibody Drug Conjugates. mAbs.

[207] Cahuzac H., Devel L. (2020). Analytical Methods for the Detection and Quantification of Adcs in Biological Matrices. Pharmaceuticals.

[208] Hernández-Jiménez J., Martínez-Ortega A., Salmerón-García A., Cabeza J., Prados J.C., Ortíz R., Navas N. (2018). Study of Aggregation in Therapeutic Monoclonal Antibodies Subjected to Stress and Long-Term Stability Tests by Analyzing Size Exclusion Liquid Chromatographic Profiles. Int. J. Biol. Macromol..

[209] Jones J., Pack L., Hunter J.H., Valliere-Douglass J.F. (2020). Native Size-Exclusion Chromatography-Mass Spectrometry: Suitability for Antibody–Drug Conjugate Drug-to-Antibody Ratio Quantitation across a Range of Chemotypes and Drug-Loading Levels. mAbs.

[210] Sun D., Gao W., Hu H., Zhou S. (2022). Why 90% of Clinical Drug Development Fails and How to Improve It?. Acta Pharm. Sin. B.

[211] Tiberghien A.C., Howard P.W., Goundry W.R.F., McCormick M., Parker J.S. (2019). An Alternative Focus for Route Design for the Synthesis of Antibody-Drug Conjugate Payloads. J. Org. Chem..

[212] Chen L., Li B., Chen Y., Lin M., Zhang S., Li C., Pang Y., Wang L. (2025). ADCNet: A Unified Framework for Predicting the Activity of Antibody-Drug Conjugates. Brief. Bioinform..

[213] Narayanan H., Dingfelder F., Condado Morales I., Patel B., Heding K.E., Bjelke J.R., Egebjerg T., Butté A., Sokolov M., Lorenzen N. (2021). Design of Biopharmaceutical Formulations Accelerated by Machine Learning. Mol. Pharm..

[214] Mariam Z., Niazi S.K., Magoola M. (2024). Unlocking the Future of Drug Development: Generative AI, Digital Twins, and Beyond. BioMedInformatics.

[215] Laurent A. (2025). Computer Vision in Pharmaceutical Quality Control: Enhancing Drug Manufacturing.

[216] Noriega H.A., Wang X.S. (2025). AI-Driven Innovation in Antibody-Drug Conjugate Design. Front. Drug Discov..

[217] Pouzin C., Gibiansky L., Fagniez N., Chadjaa M., Tod M., Nguyen L. (2022). Integrated Multiple Analytes and Semi-Mechanistic Population Pharmacokinetic Model of Tusamitamab Ravtansine, a DM4 Anti-CEACAM5 Antibody-Drug Conjugate. J. Pharmacokinet. Pharmacodyn..

[218] Lai P.-K., Fernando A., Cloutier T.K., Kingsbury J.S., Gokarn Y., Halloran K.T., Calero-Rubio C., Trout B.L. (2021). Machine Learning Feature Selection for Predicting High Concentration Therapeutic Antibody Aggregation. J. Pharm. Sci..

[219] U.S. Food and Drug Administration, Center for Drug Evaluation and Research (2023). Using Artificial Intelligence & Machine Learning in the Development of Drug and Biological Products.

[220] Hobson A.D., Xu J., Marvin C.C., McPherson M.J., Hollmann M., Gattner M., Dzeyk K., Fettis M.M., Bischoff A.K., Wang L. (2023). Optimization of Drug-Linker to Enable Long-Term Storage of Antibody-Drug Conjugate for Subcutaneous Dosing. J. Med. Chem..

[221] Wardiana A., Jones M.L., Mahler S.M., Howard C.B. (2021). Incorporation of Unnatural Amino Acid into Antibody Fragment for Creating a Stable Antibody-Drug Conjugate. Indones. J. Pharm..

[222] Kuzman D., Bunc M., Ravnik M., Reiter F., Žagar L., Bončina M. (2021). Long-Term Stability Predictions of Therapeutic Monoclonal Antibodies in Solution Using Arrhenius-Based Kinetics. Sci. Rep..

[223] Huelsmeyer M., Kuzman D., Bončina M., Martinez J., Steinbrugger C., Weusten J., Calero-Rubio C., Roche W., Niederhaus B., VanHaelst Y. (2023). A Universal Tool for Stability Predictions of Biotherapeutics, Vaccines and in Vitro Diagnostic Products. Sci. Rep..

[224] Groël S., Menzen T., Winter G. (2023). Prediction of Unwanted Crystallization of Freeze-Dried Protein Formulations Using α-Relaxation Measurements. Pharmaceutics.

